# Phyto-oxylipin mediated plant immune response to colonization and infection in the soybean-*Phytophthora sojae* pathosystem

**DOI:** 10.3389/fpls.2023.1141823

**Published:** 2023-05-12

**Authors:** Oludoyin Adeseun Adigun, Thu Huong Pham, Dmitry Grapov, Muhammad Nadeem, Linda Elizabeth Jewell, Mumtaz Cheema, Lakshman Galagedara, Raymond Thomas

**Affiliations:** ^1^ School of Science and the Environment, Boreal Ecosystems and Agricultural Sciences, Grenfell Campus, Memorial University of Newfoundland, Corner Brook, NL, Canada; ^2^ Creative Data Solution (CDS), Colfax, CA, United States; ^3^ St. John’s Research and Development Centre, Agriculture and Agri-Food Canada, St. John’s, NL, Canada; ^4^ Department of Biology/Biotron Climate Change Experimental Research Centre, Western University, London, ON, Canada

**Keywords:** Phyto-oxylipin, *Phytophthora sojae*, soybean root and stem rot, tolerance, susceptible, soybean cultivars, food security, sustainable agriculture

## Abstract

**Introduction:**

Food security is a major challenge to sustainably supply food to meet the demands of the ever-growing global population. Crop loss due to pathogens is a major concern to overcoming this global food security challenge. Soybean root and stem rot caused by *Phytophthora sojae* results in approximately 20B $US crop loss annually. Phyto-oxylipins are metabolites biosynthesized in the plants by oxidative transformation of polyunsaturated fatty acids through an array of diverging metabolic pathways and play an important role in plant development and defense against pathogen colonization and infection. Lipid mediated plant immunity is a very attractive target for developing long term resistance in many plants’ disease pathosystem. However, little is known about the phyto-oxylipin’s role in the successful strategies used by tolerant soybean cultivar to mitigate *Phytophthora sojae* infection.

**Methods:**

We used scanning electron microscopy to observe the alterations in root morphology and a targeted lipidomics approach using high resolution accurate mass tandem mass spectrometry to assess phyto-oxylipin anabolism at 48 h, 72 h and 96 h post infection.

**Results and discussion:**

We observed the presence of biogenic crystals and reinforced epidermal walls in the tolerant cultivar suggesting a mechanism for disease tolerance when compared with susceptible cultivar. Similarly, the unequivocally unique biomarkers implicated in oxylipin mediated plant immunity [10(E),12(Z)-13S-hydroxy-9(Z),11(E),15(Z)-octadecatrienoic acid, (Z)-12,13-dihydroxyoctadec-9-enoic acid, (9Z,11E)-13-Oxo-9,11-octadecadienoic acid, 15(Z)-9-oxo-octadecatrienoic acid, 10(E),12(E)-9-hydroperoxyoctadeca-10,12-dienoic acid, 12-oxophytodienoic acid and (12Z,15Z)-9, 10-dihydroxyoctadeca-12,15-dienoic acid] generated from intact oxidized lipid precursors were upregulated in tolerant soybean cultivar while downregulated in infected susceptible cultivar relative to non-inoculated controls at 48 h, 72 h and 96 h post infection by *Phytophthora sojae*, suggesting that these molecules may be a critical component of the defense strategies used in tolerant cultivar against *Phytophthora sojae* infection. Interestingly, microbial originated oxylipins, 12S-hydroperoxy-5(Z),8(Z),10(E),14(Z)-eicosatetraenoic acid and (4Z,7Z,10Z,13Z)-15-[3-[(Z)-pent-2-enyl]oxiran-2-yl]pentadeca-4,7,10,13-tetraenoic acid were upregulated only in infected susceptible cultivar but downregulated in infected tolerant cultivar. These microbial originated oxylipins are capable of modulating plant immune response to enhance virulence. This study demonstrated novel evidence for phyto-oxylipin metabolism in soybean cultivars during pathogen colonization and infection using the *Phytophthora sojae*-soybean pathosystem. This evidence may have potential applications in further elucidation and resolution of the role of phyto-oxylipin anabolism in soybean tolerance to *Phytophthora sojae* colonization and infection.

## Introduction

Soybean is an important crop grown worldwide for the purpose of high-quality, inexpensive proteins, vegetable oils, biofuels, and animal feed (Zachary et al., 2020; Mohammad Sohidul et al., 2022). The crop is cultivated on an approximately 6% of the world’s arable land, and since late 19^th^ century, the cultivated area of soybean production has the highest percentage increase more than any other major agronomic crop (Hartman et al., 2011). Recent increases in the production of soybean correspond to increases in the demand for soy protein meal and oil (Hartman et al., 2011). Therefore, further increase in soybean production is required to support the current population growth patterns and ever-changing demand for soy protein meal and oil (Mohammad Sohidul et al., 2022). However, there are a number of environmental stressors that threaten the production of soybean directly or indirectly reducing seed yields and quality (Hartman et al., 2011). Pathogen attack is one of the most devastating biotic stresses preventing the growth, development, and productivity of soybean crops worldwide (Mcdonald and Stukenbrock, 2016). Pathogens cause huge losses in terms of crop yield and quality, and consequently lead to reduction of food security and availability at global levels (Savary et al., 2019). Phytophthora root and stem rot, caused by oomycete pathogen *Phytophthora sojae* (*P. sojae*) is a significant soilborne disease of economic concern in many areas of soybean production (Giachero et al., 2022). It can destroy and kill seedlings and plants all through the growing season from early stage until harvest (Giachero et al., 2022). In the past four decades, disease management has contributed massively to plant health and food production (Nelson, 2020), but global harvests are still reduced by 10-16% due to plant diseases caused by infectious microorganisms like bacteria, viruses, nematodes and fungi (Chakraborty and Newton, 2011; Wei et al., 2019). Today, sustainable agriculture is capable of reducing the economic effects of infectious pathogens by developing disease-resistant crops using selective cross-breeding and genetic engineering to improve long-term food production and availability to meet the ever-increasing world population food security needs (Zhao et al., 2008).

Phyto-oxylipins constitute a broad class of oxygenated bioactive metabolites, believed to be involved in signaling and defense responses against pathogen attack in higher plants (Stumpe and Feussner, 2006; Adigun et al., 2020). Plant oxylipins are produced from oxidation and conversion of polyunsaturated fatty acids (PUFAs), mainly linolenic and linoleic (C18:3 and C18:2) acids, and they have been demonstrated to function in the signaling pathways that control the expression of defense-related genes such as *9-LOX*-, *13-LOX*-, and α-*DOX-1* (Howe and Schilmiller, 2002; Wu and Baldwin, 2010; García-Marcos et al., 2013). Biosynthesis of phyto-oxylipins from PUFAs *via* enzymatic processes is primarily initiated by lipoxygenases (LOXs) and *α*-dioxygenase (*α*-DOX) (Blée, 2002; Howe and Schilmiller, 2002). These lipids can also be subjected to nonenzymatic decarboxylation to form one-carbon-shortened fatty acids (FAs) and aldehydes (Granér et al., 2003). Hydroperoxides produced *via* the enzymatic action of 9-/13-LOXs can be metabolized by six major enzymatic paths: (1) to generate 9- or 13-HOD and 9- or 13-HOT through reduction *via* peroxygenase (PO) (Blée, 2002) or peroxidase activity (Brodhun et al., 2013); (2) conversion of trihydroxylated FAs into epoxy alcohols, through enzymatic action of a PO, and subsequently by an epoxide hydrolase (EH) (Blée, 2002) or through an epoxy-alcohol synthase (Brodhun et al., 2013); (3) into FA ketotrienes or ketodienes *via* dehydration through LOXs (Vollenweider et al., 2000) or through dehydrogenation of FA hydroxides by characterized enzyme (Vincenti et al., 2019); (4) into reactive hemiacetals through the activity of 9- or 13-hydroperoxide lyases (Grechkin et al., 2006; Stumpe and Feussner, 2006); (5) into divinyl ether FAs through the activity of divinyl ether synthases (DESs) (Stumpe and Feussner, 2006); and (6) into reactive allene oxides produced *via* allene oxide synthases (AOSs) (Tijet and Brash, 2002). Unstable allene oxides can undergo nonenzymatic hydrolysis producing *α*- or *γ*-ketols, or generation of cyclic compounds like cyclopentenones *via* enzymatic cyclization by allene oxide cyclases. C18 cyclopentenones, such as 12-oxo-10,15(*Z*)-phytodienoic acid undergo reduction to form cyclopentanones and are β-oxidized into short-chain compounds like jasmonic acid (JA) (Mukhtarova et al., 2020). Other oxylipins like dihydroxy FAs can be produced from C18 PUFAs through the action of the PO or EH pathways (Blée, 2002). Free fatty acids (FFAs) can also serve as substrates to produce phyto-oxylipins.

Production of oxylipins occurs constitutively in plants and as a response to various environmental stresses (Scala et al., 2018). Over 200 phyto-oxylipins have been observed so far in plants (Prost et al., 2005). Phyto-oxylipins are mainly induced during plant-pathogen interactions (Blée, 2002; Adigun et al., 2020). In fact, some phyto-oxylipins generated in defense responses against pathogen infections are antimicrobial in nature (Prost et al., 2005). Some oxylipins are described as anti-oomycete or antifungal compounds such as 13(*S*)-Hydroperoxy-9(*Z*),11(*E*),15(*Z*)-octadecatrienoic acid, 13(*S*)-Hydroxy-9(*Z*),11(*E*),15(*Z*)-octadecatrienoic acid (Granér et al., 2003), epoxy-FAs or polyhydroxylated FAs (Blée, 2002), and colnelenic acid and colneleic acid (Göbel et al., 2002). In addition, the growth of *Pseudomonas* spp. *in vitro* could be strongly inhibited by trans-2-hexenal and cis-3-hexenol (Prost et al., 2005). They are mainly understood as agents that promote resistance to pathogen attack (Christensen and Kolomiets, 2011).

Genetic studies have demonstrated the functions of α-DOX-1 and 9-LOX in the defense response of Arabidopsis and tobacco to infectious pathogen attack, likely by controlling oxidative stress and programmed cell death (PCD) (Rancé et al., 1998; De León et al., 2002). More importantly, several phyto-oxylipins generated from the activity of 9-/13-LOX were capable of initiating PCD and hypersensitivity response (HR) in some pathosystems (Cacas et al., 2005). Additionally, JA has been involved in the signaling cascade resulting in elicitation of LOX. Methyl jasmonate (MeJA) was demonstrated to trigger LOX activities and the expression of the synthesis-related genes such as *PtLOX1, PtLOX2* and *PtLOX3* (Marmey et al., 2007; Chen et al., 2015). Several pieces of evidence indicate that phyto-oxylipins play critical functions in the development of HR and disease resistance (Kovač et al., 2009; Gullner et al., 2010). However, there is a paucity of information about the function of phyto-oxylipins during fungal-plant interactions. Detailed knowledge of the molecular signaling that occurs during plant–pathogen interactions can pave the way for mechanisms of disease resistance in plants. As demonstrated in our previous studies, understanding the plant lipidome and metabolism during pathogen attack or infections is critical to elucidate their roles in susceptible or tolerant host-pathogen interactions, lipid metabolism mediated signaling, and defense responses during pathogenicity. We hypothesized that a tolerant soybean cultivar would upregulate oxylipin synthesis compared to a susceptible cultivar following *P. sojae* infection as a component of its successful mechanism used to mitigate infection by pathogens. Hence, we analyzed phyto-oxylipin compounds in the root and stem tissues of both a susceptible and a tolerant soybean cultivar to better understand the roles and induction of phyto-oxylipins in defense response during infection by *P. sojae*.

## Materials and methods

### Planting and inoculation of soybean cultivars

Seeds of susceptible (OX760-6) and tolerant (Conrad) soybean cultivars were surface sterilized using dilute sodium hypochlorite (0.5%) for 5 min (Commercial Javex Bleach; Clorox Co., Brampton, Ontario, Canada), and washed several times with distilled water (dH_2_O). Seeds were submerged for 12 h in dH_2_O and then seeded in plastic pots (195 mm diameter and 195 mm depth) containing vermiculite (#2A, Thermo-O-Rock East Inc., New Eagle, Pennsylvania) as a medium, which was then saturated with dH_2_O and the seeds were allowed to germinate. Seedlings were maintained under controlled conditions with 16 h of alternating light at 25°C and 8 h of dark at 20°C with relative humidity of 60% inside a growth chamber (Biochambers MB, Canada). Sterilized dH_2_O was applied every day to maintain the vermiculite water content from moist to slightly dry to provide optimum nutrients and moisture to seedlings. *Phytophthora sojae* virulent strain race 2 (strain P6497) was obtained from Agriculture and Agri-Food Canada (AAFC), London, Ontario, Canada. *Phytophthora sojae* was cultured and aseptically grown in darkness on 26% V8-juice agar (8.4 g agar, 1.6 g CaCO_3_, 156 mL V8-juice [Campbell Soup Company, Toronto, ON, Canada], and 440 mL dH_2_O) for 8 days. To monitor the successive events in the process of infection in roots and stems of both soybean cultivars, 8-day-old cultures of *P. sojae* were flooded with dH_2_O to produce zoospores, and then incubated overnight at 22°C. The dH_2_O was changed every 30 min for not less than 5 h. When zoospores could be observed microscopically, the concentration of zoospores was determined by adding of one drop of 0.1% wt/vol of aniline blue in lactophenol (1:1:1 85% lactic acid, phenol, and water) to 1 mL zoospore suspension; a 10 µL aliquot of this zoospore suspension was loaded onto a hemocytometer using a micropipette. The concentration of zoospores was calculated and adjusted to 1 × 10^-4^ zoospores/mL by adding deionized water. The seedlings were allowed to grow for 10 days and then carefully removed from vermiculite and washed with water to remove any remaining vermiculite from the roots. Whole seedlings from each sample were placed into 15 mL centrifuge tube containing 10 mL dH_2_O and inoculated with 0.01 mL *P. sojae* zoospore suspension, and another set of samples were mock inoculated as control without *P. sojae* zoospore suspension. There were four replications per treatment. The samples were then incubated at room temperature for periods of 48 h, 72 h and 96 h. *Phytophthora. sojae* was chosen as a test because it is an oomycete pathogen causing one of the devastating soilborne disease of economic concern in many areas of soybean production and creates serious limitations for soybean production (Sugimoto et al., 2012; Giachero et al., 2022). Furthermore, *Phytophthora sojae* is capable to destroy and kill seedlings and plants all through the growing season from early stage nearly until harvest (Giachero et al., 2022). Much is still not known concerning the etiology of this pathogen in the *soybean-P. sojae* pathosystem specifically related to plant tolerance.

For determination of oxidized glycerolipids and histochemical analysis in both soybean cultivars, the seeds and fungal cultures were prepared as described above. Agar disks containing cultures of *P. sojae* strain P6497 were cut and fitted into the bottom of wax-paper cups (top diameter 8.5 cm by 15 cm deep: Merchants Paper Company, Windsor, ON, Canada). These cups were then filled up with medium-grade vermiculite, drainage holes were created in the bottom of the cups, and six seeds were planted in each of four replications cups containing vermiculite. For non-inoculated controls, agar disks without *P. sojae* were used. Seedlings were maintained in the growth chamber at the same conditions as described above for 10 days. Six seedlings from each soybean cultivar were inoculated with *P. sojae* in a cup and another six from each soybean cultivar were mock-inoculated using only sterile V8-juice agar disks to serve as a control. Beginning four days after seeding, seedlings were watered daily using one-quarter-strength Knop’s solution (Thomas et al., 2007). The entire plants were harvested 10 days after germination and kept at -80°C until analysis. Figure representations of experimental set-up supplementing the detail of the growth chamber are demonstrated in **Figures S1**, **S2**.

### Sample preparation for scanning electron microscopy

Soybean roots were collected from non-inoculated and inoculated tolerant and susceptible soybean cultivars. The plant samples were rinsed with distilled water before further analysis. Free-hand cross sections of the soybean root were cut to a length of approximately 5 mm using a razor blade. Thin sections were mounted using colloidal graphite adhesive (Permatex, Canada, Incorporated) into aluminum stubs. The samples were unveiled to a temperature of -4.9°C on a Peltier cooling stage to reduce differences in structure, while in the vacuum chamber. The images of the soybean roots were obtained using an environment scanning electron microscope (ThermoFisher Quattro S with ESEM), to study the morphological properties of soybean roots infected with *P. sojae*. High-resolution images were obtained from 9-10 mm to 5-100 µm (magnification 788-8000X with the pressure 50-428 Pa) (Tihlaříková et al., 2013; Zhang et al., 2019).

### Extraction of oxidized glycerolipids and primary oxylipins from soybean tissues

Lipid extraction was performed by incubating the entire soybean seedlings from both experiments in hot isopropanol for 10 min and then separating the root and stem, and 100 mg of tissue from each sample were collected for lipid extraction according to previously published methods (Nadeem et al., 2020; Adigun et al., 2021). The extracted lipids from four replications were injected into ultra high-performance liquid chromatography coupled with C30-reversed phase chromatography and ionized using heated electrospray ionization linked to high resolution accurate mass tandem mass spectrometer (UHPLC-C30RP-HES-HRAM-MS/MS) to separate the oxidized glycerolipids.

For the extraction of primary oxylipins, the soybean samples from the zoospore inoculation were weighed into 100 mg subsamples. The samples were placed into 2 mL glass centrifuge tubes containing 300 µL of 10% glycerol in water and treated immediately with 5 µL of 10 mg/mL butylated hydroxyl toluene (BHT) dissolved in ethanol. Each sample was then spiked with 20 µL of a suitable deuterium-labeled internal standard with a concentration of 500 ng/mL in ethanol. The sample volume was made up to 3 mL with 25% aqueous acetonitrile in a centrifuge tube before the tube was placed in ice and homogenized using a probe tissue homogenizer. The extracted solvent mixtures were centrifuged for 10 mins at 5500 rpm and 4°C to obtain the supernatants. The extraction of plant samples was performed using solid phase extraction (SPE) with water using an OASIS MAX SPE column (3 cc, Vac Cartridge, 30 µM particle size, part number 186000367) for concentrating phyto-oxylipins. The column was initially conditioned with 3 mL acetonitrile, and subsequently with 3 mL of 25% aqueous acetonitrile. Then, the entire supernatant from the centrifuged sample was loaded onto the SPE column and the SPE column was gently washed with 3 mL of 25% aqueous acetonitrile, followed by 3 mL acetonitrile. Oxylipins were eluted from the column into a glass vial containing 200 µL of 10% glycerol in methanol with 1.3 mL of 1% formic acid in acetonitrile (1: 99 v/v). The eluent was dried under nitrogen at 40°C until only the glycerol remained. The dried eluates were then reconstituted in 60 µL of a methanol:acetonitrile (1:1v/v) solution and vortexed thoroughly. Afterwards, the eluates were filtered using 0.1 µM Amicon Ultrafree-MC Durapore PVDF filter (pore-size 0.1 µM; Millipore, Bedford, MA). Finally, 3 µL of each sample were injected and oxylipins resolved using ultra high-performance liquid chromatography containing a C18 bridge ethylene hybrid column coupled to heated electrospray ionization high resolution accurate mass tandem mass spectrometry (UHPLC-C18-BEH-HESI-HRAM-MS/MS; Q Exactive, ThermoFisher Scientific, ON, Canada). Four replications per sample were used for analysis.

### Analysis of oxidized glycerolipids and primary oxylipins from susceptible and tolerant soybean cultivars

Analysis of oxidized glycerolipids induced in the root and stem of both soybean cultivars were performed using UHPLC-C30RP-HESI-HRAM-MS/MS. Detailed chromatographic conditions for analysis were as previously described (Nadeem et al., 2020; Adigun et al., 2021).

For primary oxylipin analysis, 100 µL extracts were introduced into automated Dionex UltiMate 3000 UHPLC system and the auto sampler was cooled to a temperature of 10°C. Chromatographic separation was performed on an Acquity UHPLC-BEH, 1.7 µM, 2.1 x 100 mm C18 column using a flow rate of 0.2 mL/min at 30°C during a 26 min gradient (0–3.5 min from 15% B to 33% B, 3.5–5.5 min to 38% B, 5–7 min to 42% B, 7–9 min to 48% B, 9–15 min to 65% B, 15–17 min to 75% B, 17–18.5 min to 85% B, 18.5–19.5 min to 95% B, from 19.5 to 21 min to 15% B, and from 21–26 min 15% B). Mobile phase A consisted of aqueous 0.1% acetic acid, and mobile phase B was 90:10 v*/*v acetonitrile/isopropyl alcohol. A Q-Exactive orbitrap mass spectrometer was used and the data acquired in the negative mode at temperature 100°C, capillary spray voltage 3.0 kV, capillary temperature 300°C, S-lens RF level 30 V, sheath gas temperature 350°C, auxiliary gas setting 2, energy: 32.5 (stepped collision energy 30 and 35, arbitrary unit). The full scan mode at 70,000 m/z resolution, top-10 data dependent MS/MS at 35,000 m/z resolution, 1 m/z isolation window and 1e6 automatic gain control target was utilized. The equipment was calibrated externally to 1 ppm using tuning solution (Pierce™ LTQ Velos ESI Positive Ion Calibration Solution and Pierce™ Negative Ion Calibration Solution) purchased from Thermo Scientific (Waltham, MA, USA).

### Oxylipin network mapping from susceptible and tolerant soybean cultivars

To obtain comprehensive knowledge from a systems biology perspective of how susceptible and tolerant soybean cultivars biosynthesize phyto-oxylipins as part of defense strategy against pathogen invasion, phyto-oxylipins that exhibited significant changes in relative concentrations as a result of the treatment were visualized within oxylipin structural similarity networks. Regularized oxylipin correlation networks were calculated and visualized to obtain insights into alterations between soybean cultivars. Networks were separately calculated for root and stem tissue at 48 h, 72 h and 96 h post inoculation. Correlations between oxylipins were calculated using high-dimensional undirected graph estimation method (Zhao et al., 2012). Relationships between lipids were estimated based on Meinshausen-Buhlmann graph estimation and the stability approach to regularization selection to identify conditionally independent oxylipin-oxylipin connections (Meinshausen and Bühlmann, 2006). The relationships were created between both soybean cultivars and experimental groups inoculated at 48 h, 72 h and 96 h time points. The regularization lambda for root and stem network at time points 48 h, 72 h and 96 h were specified at 0.46, 0.28 and 0.34 for root and 0.46, 0.17 and 0.13 for stem networks respectively. Mapped networks were created to visualize changes in relationships between oxylipins and experimental differences. Linear models were built to identify significant interactions between changes in oxylipins between cultivars and inoculation status at each individual time point of 48 h, 72 h and 96 h (Team, 2014). Significant interactions were identified based on false discovery adjusted p-values (pFDR) < 0.05. Significant changes in oxylipin abundances between pairwise comparisons of cultivar and inoculation groups were evaluated based on Tukey’s Honestly Significant Difference (HSD) method. Significant changes between groups were identified based on HSD-adjusted p < 0.05. Magnitude and direction (positive or negative) of the relationships were determined based on the Spearman correlations (pFDF < 0.05). Significant interactions between cultivar and inoculation were identified based on linear model pFDR < 0.05 and HSD p-values < 0.05. Cytoscape was used to render oxylipin-oxylipin interactions (Shannon et al., 2003) and show all pairwise differences between cultivar and inoculation groups. Network node colors was used to show magnitude (size) and direction (color) of fold changes and will be reported as means for all experimental groups compared to the following references: tissue type (root or stem), cultivar (susceptible or resistant), and treatment type (inoculated or control).

### Data analysis

To determine the effects of plant-pathogen interaction on phyto-oxylipin induction in the root and stem of susceptible (OX760-6) and tolerant (Conrad) soybean cultivars, partial least squares-discriminant analysis (PLS-DA), and heatmap analysis were conducted with XLSTAT (Premium 2017, Version 19.5, Addinsoft). Results are presented as average ± standard error unless noted otherwise. The means with significant differences were compared using Fisher’s Least Significant Difference (LSD), *a* = 0.05. SigmaPlot 13.0 software (Systat Software Inc., San Jose, CA) was used for figure preparation. Linear models were built to identify significant interactions between changes in oxylipins due to tissue, cultivar and treatment at each individual inoculation time point. Significant interactions were identified based on false discovery adjusted p-values (pFDR) < 0.05. Pairwise changes between all groups (tissue × cultivar × treatment) were evaluated based on Tukey’s HSD method. Significant changes between groups were identified based on HSD-adjusted *p* < 0.05. Note that all analyses were done separately for each time point due to observed non-linear trends in lipids changes over time.

## Results

### Histological alterations in the root of susceptible and tolerant soybean cultivars infected with *Phytophthora sojae*

*Phytophthora sojae* is a fungal-like oomycete pathogen that lives dormant for many years in soil in the form of encysted oospores without a host until the conditions are suitable for regeneration (Gaulin et al., 2018). When soybean is infected by oomycete *P. sojae*, the soybean stem appears water-soaked and changed to red-brown, and the infected plant results in wilting and death (Sugimoto et al., 2012). *Phytophthora sojae* generates motile zoospores from infected soybean tissues or activated zoospores following breaking dormancy after encystment are capable to initiate further disease cycles (Sugimoto et al., 2012).

**Figure 1:**
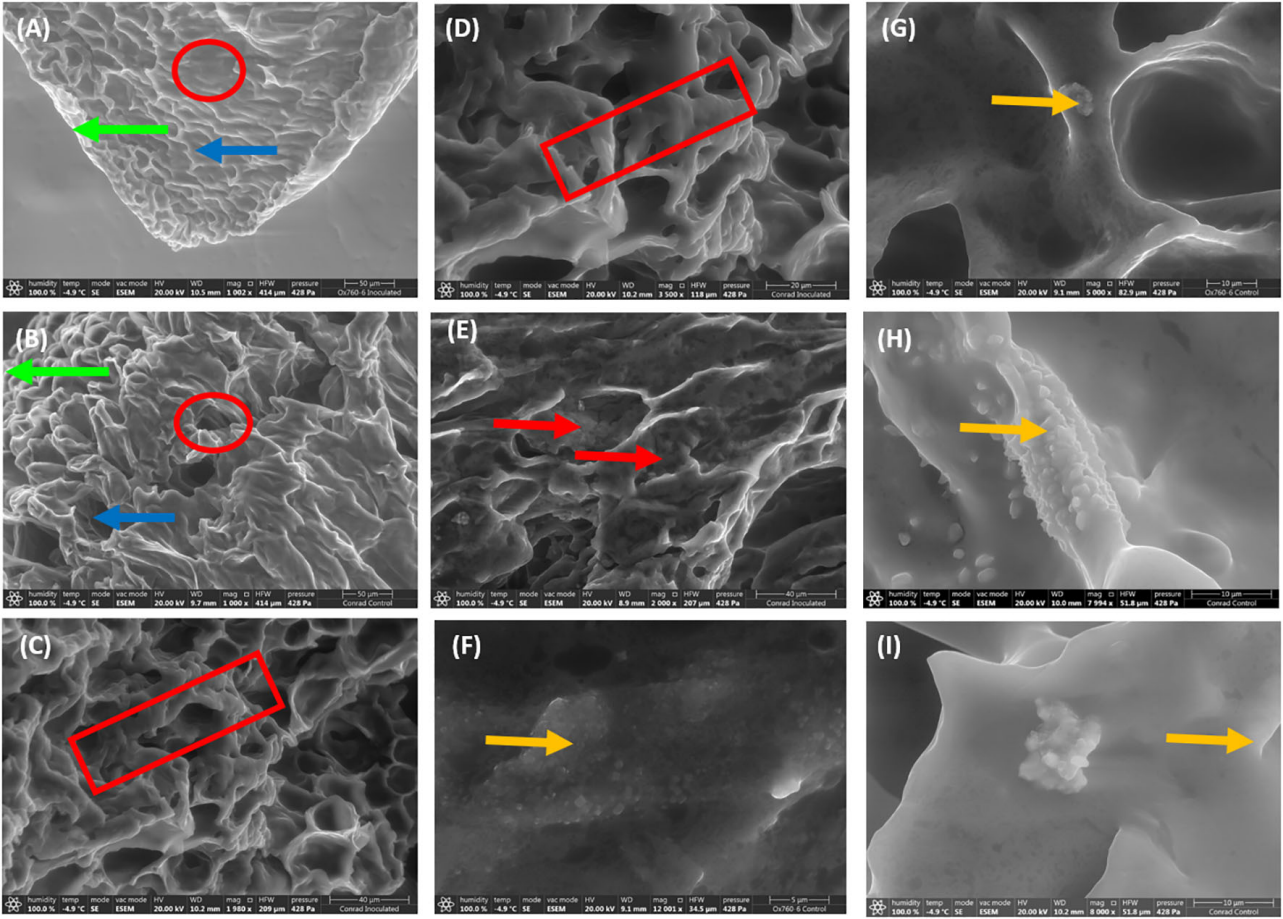
Scanning electron microscopy demonstrating unique features in soybean root morphology. The root epidermis, cortex and vascular cylinder with low magnification indicated by green arrow, blue arrow and red circle respectively (**A**, **B**; 50 µm). High magnification of root tissues showing contraction of root indicated by red rectangular (**C**, **D**; 40 and 20 µm), the presence of occluding materials indicated by red arrow in the root cortex (**E**; 40 µm), and the presence and distribution of biogenic crystals indicated by yellow arrow within the vascular cylinder of soybean root (**F**–**I**; 5-10 µm).

To understand the root morphology of soybean cultivars and how changes occur morphologically in the root during interaction with *P. sojae* governing tolerance. Scanning electron microscopy (SEM) was used to obtain detailed structural images of soybean root morphology revealing important altered features in the epidermis, cortex and vascular cylinder of the root at 50 µm magnification (**Figures 1A, B**), 40 µm (**Figure 1C**) and 20 µm (**Figure 1D**). At these various magnifications we observed the following features in the root tissues: the presence of occluding materials in the root cortex at 40 µm magnification (**Figure 1E**), distribution of biogenic crystals of various morphologies and sizes within the vascular cylinder of soybean root at 5 µm (**Figure 1F**) and at 10 µm magnifications (**Figures 1G–I**). The histological features in soybean root morphology after infection were differentiated in term of low magnification and high magnification.

**Figure 2:**
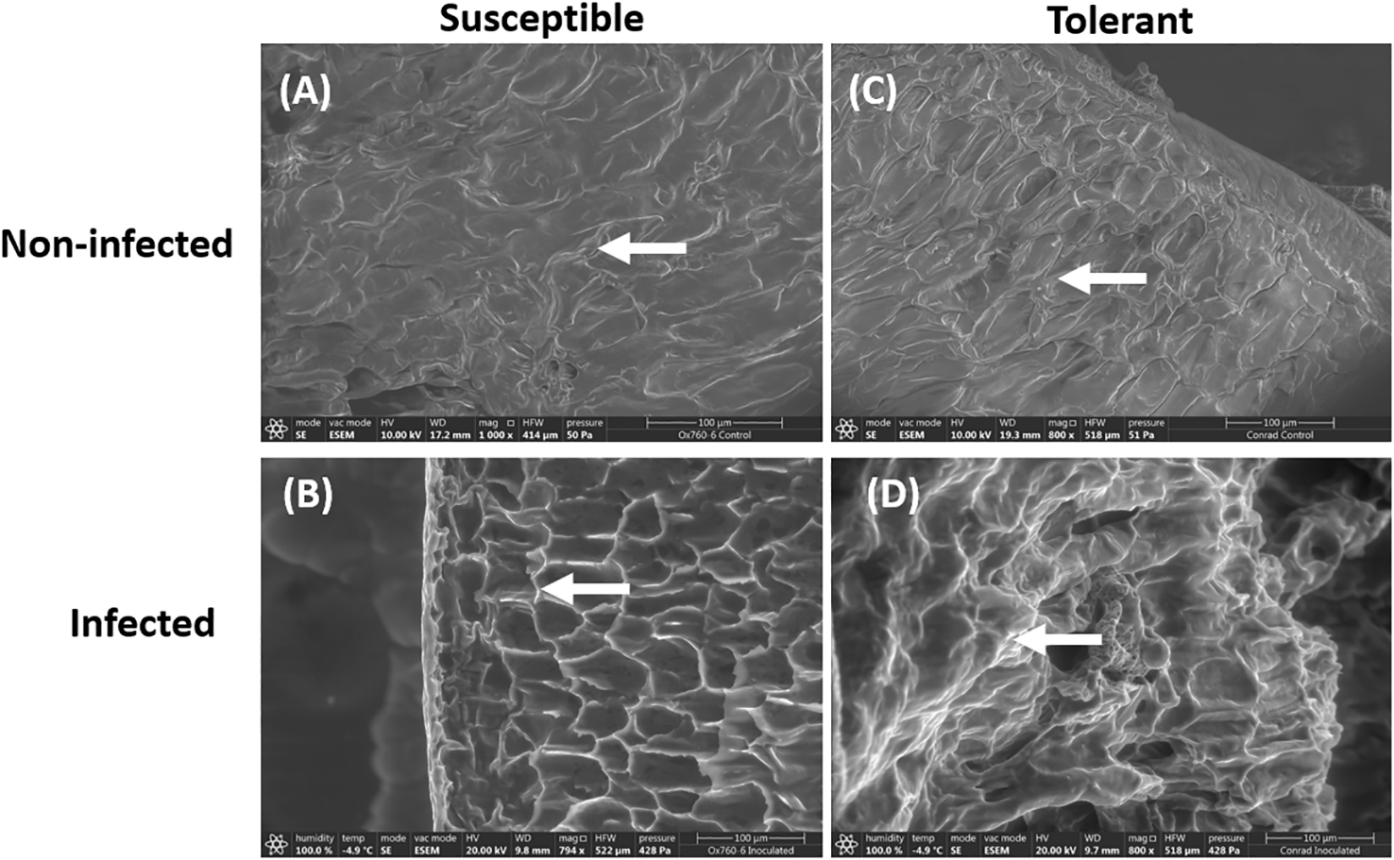
Scanning electron microscopy showing the root epidermal walls in susceptible (OX760-6) and tolerant (Conrad) soybean cultivars when inoculated with *P. sojae.* **(A)** The epidermal walls of the non-infected susceptible soybean cultivar, **(B)** Epidermal walls of the infected susceptible soybean cultivar, **(C)** Epidermal walls of the non-infected tolerant soybean cultivar, **(D)** Epidermal walls of the infected tolerant soybean cultivar. White arrows denote epidermal walls of the roots. The epidermal cells appear to be more regular in shape and clearly visible in the tolerant cultivar than in the susceptible cultivar. Bars: **(A–D)** 100 µm.

Similarly, only the epidermal walls of the inoculated tolerant cultivar were visibly infected. In contrast, the inoculated susceptible cultivar had presentation of infection traversing to the cortex showing signs of being highly infected, where the cortex appeared to be severely damaged (**Figures 2B, D**). In the non-inoculated roots of both soybean cultivars, the cortical cells were observed to be closely packed without any damage (**Figures 2A, C**).

**Figure 3:**
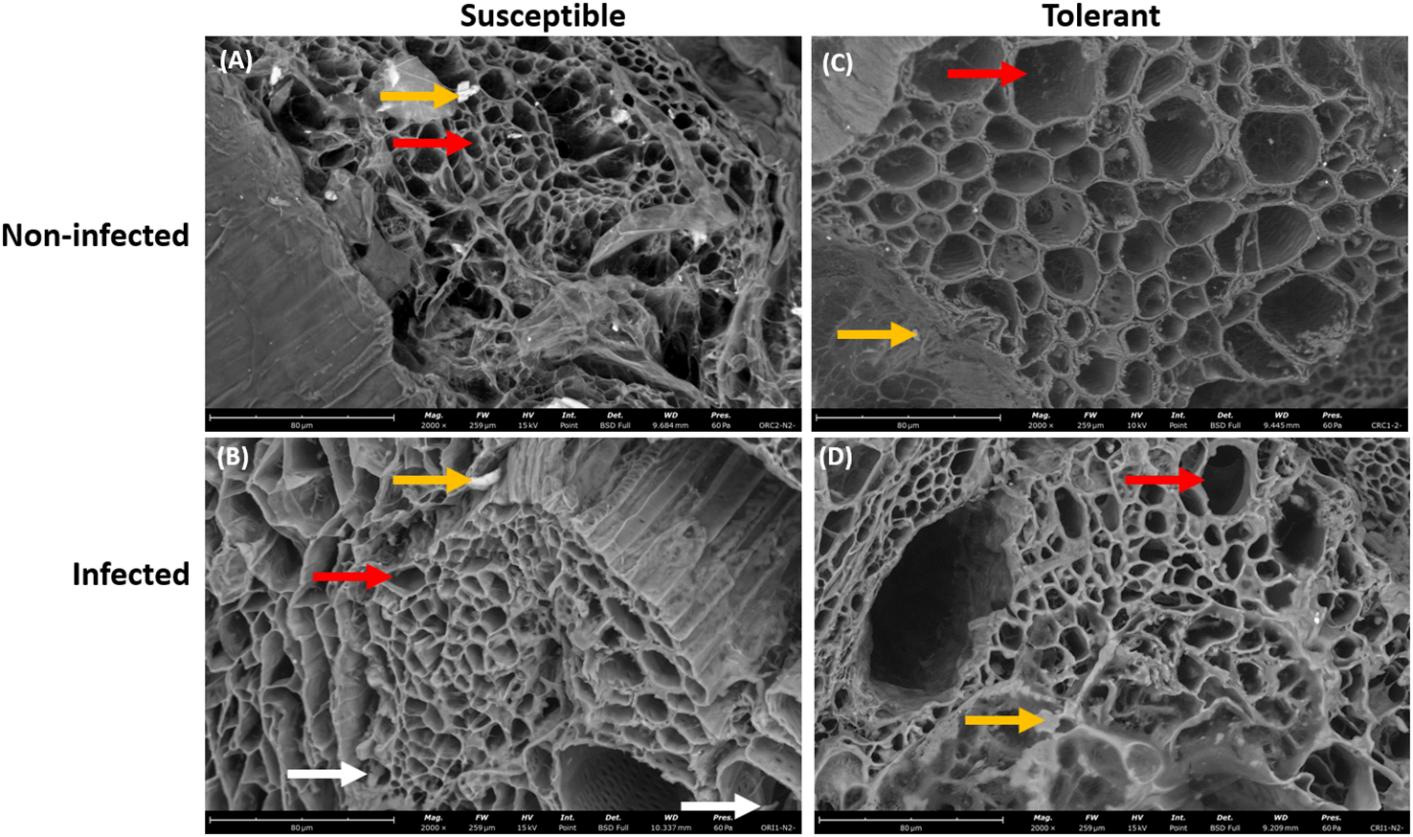
Scanning electron microscopy showing the root xylem vessels of susceptible and tolerant soybean cultivars when inoculated with *P. sojae.* **(A)** Vascular cylinder of the non-infected susceptible soybean cultivar, **(B)** Vascular cylinder of the infected susceptible soybean cultivar, **(C)** Vascular cylinder of the non-infected tolerant soybean cultivar, **(D)** Vascular cylinder of the infected tolerant soybean cultivar. The root of susceptible cultivar contains small vascular cylinder while the root of tolerant cultivar contains large vascular cylinder, denoted by red arrows. White arrows denote the presence of hyphae within the vascular cylinder and yellow arrows denote the presence of vermiculite. Bars: **(A–D)** 80 µm.

In addition, the root of the susceptible soybean cultivar contained small vascular cylinder while the root of the tolerant soybean cultivar had large vascular cylinder (**Figures 3A–D**). The walls of these root cells were intact indicating that these cells were living when inoculated (**Figures 3A–C**). After 10 days of seedling growth, hyphae had generally penetrated the epidermis and the outer layers of cortical cells of both soybean cultivars. The hyphae were able to colonize the vascular cylinder of the susceptible cultivar only.

**Figure 4:**
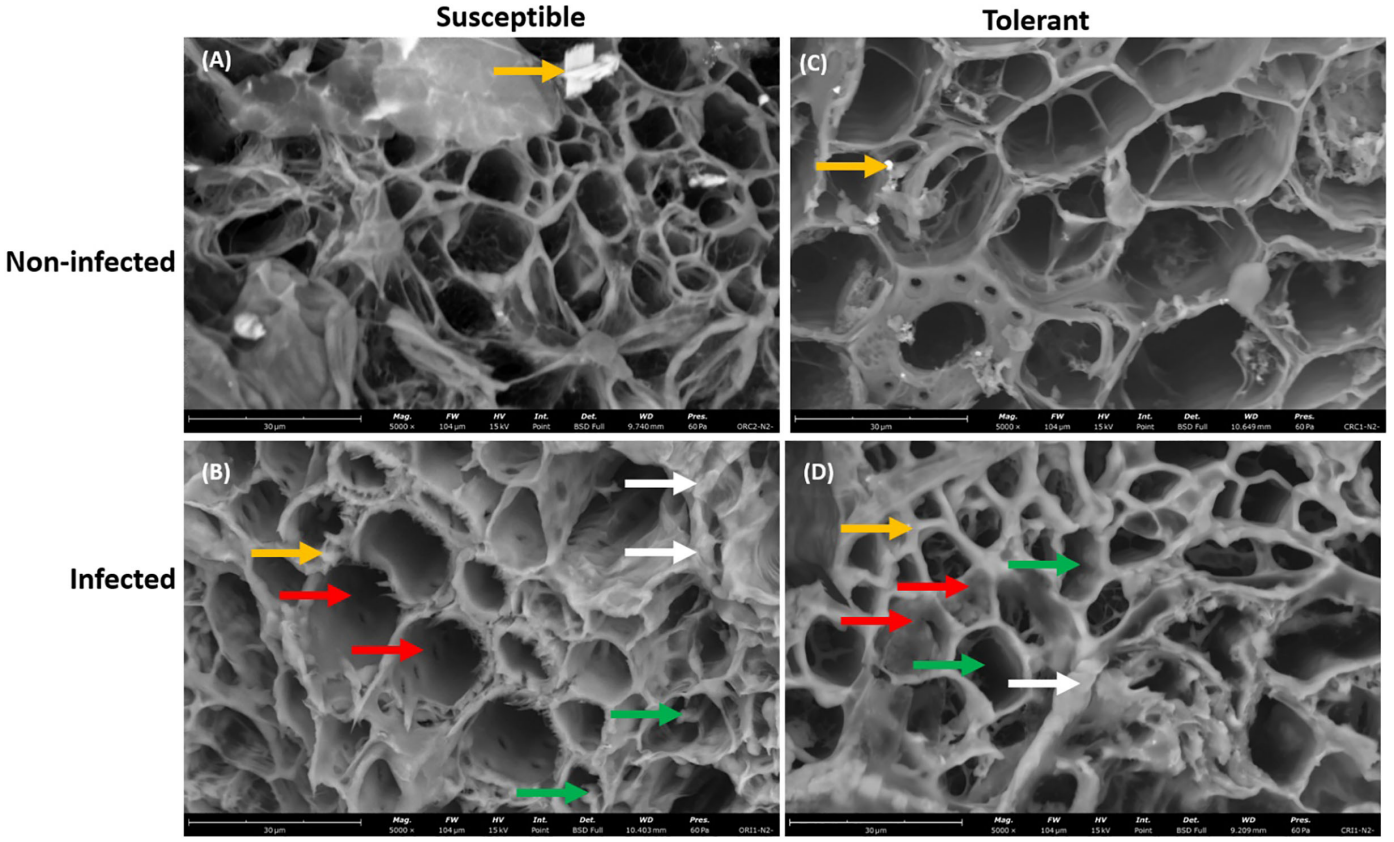
Scanning electron microscopy showing the large view of root vascular cylinder of susceptible and tolerant soybean cultivars when inoculated with *P. sojae.* **(A)** Cross section of the non-infected susceptible soybean cultivar, **(B)** Cross section of the infected susceptible soybean cultivar, **(C)** Cross section of the non-infected tolerant soybean cultivar, **(D)** Cross section of the infected tolerant soybean cultivar. The vascular cylinder of the susceptible cultivar contained hyphae while the vascular cylinder of tolerant cultivar shows the presence of occluding materials. Green arrows denote hyphae, white arrows denote the presence of unknown debris in cortical cells, red arrows denote the presence of occluding materials and yellow arrows denote the presence of vermiculite. Bars: **(A–D)** 30 µm.

Furthermore, the presence of *P. sojae* hyphae was more pronounced in the vascular cylinder of the susceptible cultivar than in the vascular cylinder of the tolerant cultivar, which is denoted with green arrow (**Figures 4B–D**). In addition, occluding materials were observed in the vascular cylinder of the tolerant cultivar but not observed in the vascular cylinder of the susceptible cultivar, which is denoted with red arrow (**Figures 4B–D**), and the presence of unknown debris in cortical cell was more pronounced in the infected susceptible cultivar than in the infected tolerant cultivar (**Figures 4B–D**). The presence of the vermiculite medium was observed in the roots of both non-infected and infected soybean cultivars (**Figures 4A–D**).

### Phyto-oxylipin profiling in susceptible and tolerant soybean cultivars in response to *P. sojae* infection

In both susceptible and tolerant soybean cultivars, approximately 30 oxylipins were identified irrespective of treatment. Primary oxylipins and oxidized glycerolipids with the highest influential loading were determined using PLS-DA. Nine primary oxylipins were identified from the root and stem of both soybean cultivars and they were classified according to their pathway of origin (either LOX, AOS, or cytochrome P450). Detected oxylipins originating from LOX were: 10(E),12(E)-9-hydroperoxyoctadeca-10,12-dienoic acid (9-HpODE), 10(E),12(Z), 13S-hydroxy-9(Z),11(E),15(Z)-octadecatrienoic acid (13-HOTrE), and 12S-hydroperoxy 5(Z),8(Z),10(E),14(Z)-eicosatetraenoic acid (12(S)-HpETE), 9-oxo-10E,12Z,15Z-octadecatrienoic acid (9-KOTrE) and (9Z,11E)-13-Oxo-9,11-octadecadienoic acid (13-KODE). Those identified from the AOS pathway were: 12-oxophytodienoic acid (12-OPDA); and while the following were from the cytochrome P450 pathway: (12*Z*,15*Z*)-9,10-dihydroxyoctadeca-12,15-dienoic acid (9, 10-DiHODE), (*Z*)-12,13-dihydroxyoctadec-9-enoic acid (12,13-DiHOME), and (4*Z*,7*Z*,10*Z*,13*Z*)-15-(3-((*Z*)-pent-2-enyl)oxiran-2-yl)pentadeca-4,7,10,13-tetraenoic acid (16,17-EpDPE) (**Tables 1**, **2**). Chromatograms and mass spectrums of oxylipins observed in tolerant and susceptible soybean roots and stems in response to *P. sojae* infection are showing in **Figure S3**. A chromatogram showing the separation of primary oxylipins from inoculated root of both soybean cultivars is presented in **Figure S3A**. The extracted ion chromatogram (XIC) of m/z 293.21, 313.24 and 335.22 precursor ions of the selected primary oxylipins is shown in **Figure S3B**. The MS^2^ spectrum of *m/z* 293.21 recognized as 13-KODE is presented in **Figure S3C**, the MS^2^ spectrum of *m/z* 313.22 recognized as 12,13-DiHOME is presented in **Figure S3D**, and the MS^2^ spectrum of *m/z* 335.22 recognized as 12(S)-HpETE is presented in **Figure S3E**; these account for some of the major primary oxylipins identified in the tissues of both soybean cultivars. From our previous lipid metabolism studies of soybean cultivars challenged with *P. sojae*, we have generated a list of oxidized glycerolipids that could serve as substrates for biosynthesis of primary oxylipins.

**Table 1:** Primary oxylipins (nmol) induced in the root of soybean cultivars following inoculation with *P. sojae*.

Oxylipin pathway	Oxylipin	Relative abundance (nmol)											
		48- h				72-h				96-h			
		ORC	ORI	CRC	CRI	ORC	ORI	CRC	CRI	ORC	ORI	CRC	CRI
Lipoxygenase	9-HpODE*	45401.96^b^	19235.13^c^	3232.84^d^	60002.61^a^	44827.97^b^	0.00^d^	3308.41^d^	0.00^d^	44697.89^b^	2.67^d^	3168.44^d^	0.00^d^
	13-HOTrE*	740.27^b^	779.18^b^	84.27^c^	1615.88^a^	753.39^b^	123.22^c^	85.52^c^	530.84^b^	746.61^b^	160.55^c^	81.30^c^	1807.62^a^
	12(S)-HpETE*	0.27^b^	254.80^a^	0.35^b^	0.37^b^	0.52^b^	264.63^a^	0.58^b^	0.37^b^	0.50^b^	251.89^a^	0.11^b^	0.31^b^
	9-KOTrE*	17879.31^a^	14914.77^b^	764.41^e^	12639.78^b^	18380.18^a^	3527.42^d^	758.20^e^	7829.71^c^	18377.79^a^	2315.78^e^	761.83^e^	15073.73^b^
	13-KODE*	10103.22^b^	7810.54^c^	1321.30^d^	16099.55^a^	10042.41^b^	2122.81^d^	1330.11^d^	5622.89^c^	10042.41^b^	2379.03^d^	1318.21^d^	16051.70^a^
Allene oxide synthase	12-OPDA*	1050.02^c^	2351.15^b^	214.08^c^	1984.07^b^	1072.48^c^	306.58^c^	219.00^c^	1358.62^c^	1066.01^c^	265.42^c^	212.16^c^	6824.43^a^
Cytochrome P450	9,10-DiHODE*	54929.60^b^	58177.16^b^	6063.29^e^	65571.54^b^	55481.44^b^	7123.79^e^	6122.00^e^	30229.56^c^	55539.04^b^	12852.18^d^	6054.92^e^	98027.91^a^
	12,13-DiHOME*	2218.28^b^	2755.52^b^	326.19^e^	2395.72^b^	2032.80^b^	431.09^e^	323.49^e^	1289.37^c^	2024.81^b^	759.33^d^	328.39^e^	3918.76^a^
	16,17-EpDPE*	0.02^d^	5161.15^c^	0.52^d^	0.34^d^	0.45^d^	6357.31^b^	0.18^d^	0.53^d^	0.25^d^	7519.81^a^	0.72^d^	0.30^d^

Summary of primary oxylipin (nmol) in the roots of both soybean cultivars. Data presented are means ± standard errors for four sample replicates. Means in the same row accompanied by different superscripts represent significance differences (*) between the treatments, consisting of susceptible root control (ORC) and susceptible root inoculated (ORI); and tolerant root control (CRC) and tolerant root inoculated (CRI) from 10-day old plants following a post-inoculation period of 48 h, 72 h or 96 h. Significance level assessed at α < 0.05. The oxylipins detected were 10(E), 12(E)-9-hydroperoxyoctadeca-10,12-dienoic acid (9-HpODE), 10(E),12(Z), 13S-hydroxy-9(Z),11(E),15(Z)-octadecatrienoic acid (13-HOTrE), and 12S-hydroperoxy-5(Z),8(Z),10(E),14(Z)-eicosatetraenoic acid (12(S)-HpETE), 15(Z)-9-oxo-octadecatrienoic acid (9-KOTrE) and (9Z,11E)-13-Oxo-9,11-octadecadienoic acid (13-KODE), 12-oxophytodienoic acid (12-OPDA), (12Z,15Z)-9,10-dihydroxyoctadeca-12,15-dienoic acid (9, 10-DiHODE), (Z)-12,13-dihydroxyoctadec-9-enoic acid (12,13-DiHOME), and (4Z,7Z,10Z,13Z)-15-[3-[(Z)-pent-2-enyl]oxiran-2-yl]pentadeca-4,7,10,13-tetraenoic acid (16,17-EpDPE).

**Table 2:** Primary oxylipins (nmol) induced in the stem of soybean cultivars following inoculation with *P. sojae*.

Oxylipin pathway	Oxylipin	Relative abundance (nmol)											
		48- h				72-h				96-hr			
		OSC	OSI	CSC	CSI	OSC	OSI	CSC	CSI	OSC	OSI	CSC	CSI
Lipoxygenase	9-HpODE*	104843.20^a^	52336.91^b^	6.88^c^	0.00^c^	105792.80^a^	0.00^c^	8.47^c^	0.00^c^	104642.24^a^	9.67^c^	6.67^c^	52456.14^b^
	13-HOTrE*	2624.07^a^	2337.83^a^	8.35^c^	1417.53^b^	2671.72^a^	744.03^b^	7.56^c^	964.44^b^	2527.26^a^	4.36^c^	8.44^c^	2085.09^a^
	12(S)-HpETE*	0.39^d^	32238.70^c^	0.69^d^	0.37^d^	0.64^d^	48119.54^b^	0.71^d^	0.31^d^	0.59^d^	87843.49^a^	0.29^d^	0.88^d^
	9-KOTrE*	54403.13^a^	56014.66^a^	17.52^d^	10710.23^c^	53573.05^a^	26098.06^b^	16.95^d^	8448.08^c^	55636.58^a^	0.00^d^	19.41^d^	29448.44^b^
	13-KODE*	39289.67^a^	34647.07^a^	34.84^c^	18152.65^b^	40906.82^a^	15947.95^b^	33.13^c^	13203.10^b^	38933.45^a^	9.80^c^	35.42^c^	30887.31^a^
Allene oxide synthase	12-OPDA*	5209.15^c^	4683.41^c^	17.43^e^	8482.57^b^	5158.26^c^	1751.61^d^	16.12^e^	4358.54^c^	5250.67^c^	0.00^e^	20.83^e^	13173.55^a^
Cytochrome P450	9,10-DiHODE*	135559.71^a^	152625.22^a^	64.04^c^	51317.76^b^	135578.00^a^	31076.93^b^	61.22^c^	38868.45^b^	134435.30^a^	6.48^c^	64.53^c^	147879.60^a^
	12,13-DiHOME*	6514.48^a^	7361.51^a^	10.21^d^	4128.60^b^	6011.53^a^	2179.51^c^	8.04^d^	4379.30^b^	7502.90^a^	6.24^d^	10.14^d^	7845.75^a^
	16,17-EpDPE*	0.59^d^	19069.21^b^	0.22^d^	0.99^d^	0.60^d^	16544.95^c^	0.58^d^	0.76^d^	0.46^d^	72849.52^a^	0.27^d^	0.42^d^

Summary of primary oxylipin (nmol) in the stems of both soybean cultivars. Data represented are means ± standard errors for four sample replicates. Means in the same row accompanied by different superscripts represent significance differences (*) between the treatments, consisting of susceptible stem control (OSC) and susceptible stem inoculated (OSI); and tolerant stem control (CSC) and tolerant stem inoculated (CSI) from 10-day old plants following post inoculation period of 48 h, 72 h or 96 h. Significance level assessed at α < 0.05. The oxylipins detected were 10(E),12(E)-9-hydroperoxyoctadeca-10,12-dienoic acid (9-HpODE), 10(E),12(Z), 13S-hydroxy-9(Z),11(E),15(Z)-octadecatrienoic acid (13-HOTrE), and 12S-hydroperoxy-5(Z),8(Z),10(E),14(Z)-eicosatetraenoic acid (12(S)-HpETE), 15(Z)-9-oxo-octadecatrienoic acid (9-KOTrE) and (9Z,11E)-13-Oxo-9,11-octadecadienoic acid (13-KODE), 12-oxophytodienoic acid (12-OPDA), (12Z,15Z)-9,10-dihydroxyoctadeca-12,15-dienoic acid (9, 10-DiHODE), (Z)-12,13-dihydroxyoctadec-9-enoic acid (12,13-DiHOME), and (4Z,7Z,10Z,13Z)-15-[3-[(Z)-pent-2-enyl]oxiran-2-yl]pentadeca-4,7,10,13-tetraenoic acid (16,17-EpDPE).

The 12 oxidized glycerolipids observed in the root of both soybean cultivars included PC36:6+2O, PC36:5+2O, PE38:6+O, PE38:6+2O, PA34:3+O, PI28:3+2O, TG50:3+O, TG52:6+O, TG54:2+O, TG54:8+2O, TG54:8+3O and TG54:6+Ox (**Table 3**), and the 13 oxidized glycerolipids in the stem of both cultivars included (PC36:6+2O, PC36:5+2O, PE38:6+O, PE38:6+2O, PA34:3+O, TG54:8+2O, TG54:8+3O, TG52:6+O, TG54:9+O, TG60:9+5O, TG60:8+5O, TG60:10+6O and TG54:2+Ox (**Table 4**). These oxidized glycerolipids were generally upregulated in the tolerant soybean cultivar compared to susceptible soybean cultivar when challenged with pathogen and this may be one of defense mechanisms used by tolerant soybean cultivar. The resistant cultivar appears to be unable to catabolize these oxylipins compared to the tolerant cultivar. As such there are high bioaccumulations of pathogen derived oxylipins in the susceptible cultivar limiting the susceptible plants in their ability to activate its immune response to circumvent colonization and infection. The chromatogram showing glycerolipids in the inoculated stem of both soybean cultivars is presented in **Figure S3F**. The XIC of precursor ions *m/z* 685.44, 669.45 in negative ion mode and *m/z* 884.73 and 868.74 in the positive ion mode show the oxidized and unoxidized glycerolipids observed in soybean roots (**Figure S3G**), the MS^2^ spectra of *m/z* 685.44 and 669.45 (M-H)^-^ precursor ions showing the presence of Ox-PA (PA16:0/18:3+O) in addition to the unoxidized PA 16:0/18:3 are presented in **Figures S3H, I**, and the MS^2^ spectra of *m/z* 884.73 and 868.74 (M+NH4)^+^ precursor ions showing the presence of Ox-TG (TG 16:0/18:3/18:3+O) in addition to the unoxidized TG 16:0/18:3/18:3 are presented in **Figures S3J, K**.

**Table 3:** Oxidized glycerolipids (nmol) present in the root of soybean cultivars following inoculation with *P. sojae*.

Lipid class	Oxidized glycerolipids	Relative abundance (nmol)			
		ORC	ORI	CRC	CRI
Phosphatidylcholine	PC36:6+2O*	9.65 ± 0.88^c^	12.47 ± 0.61^b^	0.00 ± 00^d^	18.02 ± 0.10^a^
	PC36:5+2O*	20.10 ± 0.78^b^	16.58 ± 0.46^c^	0.00 ± 00^d^	23.34 ± 0.21^a^
Phosphatidylethanolamine	PE38:6+O*	15.39 ± 0.28^b^	25.23 ± 0.45^a^	0.00 ± 00^d^	8.58 ± 0.18^c^
	PE38:6+2O*	9.65 ± 0.88^b^	11.97 ± 0.39^a^	0.00 ± 00^d^	8.79 ± 0.40^c^
Phosphatidic acid	PA34:3+O*	10.67 ± 0.25^a^	6.48 ± 0.38^b^	0.00 ± 00^c^	10.85 ± 0.26^a^
Phosphatidylinositol	PI28:3+2O*	3.60 ± 0.21^a^	3.06 ± 0.13^a^	0.00 ± 00^b^	0.36 ± 0.02^b^
Triacylglycerol	TG50:3+O*	7.63 ± 0.17^b^	12.81 ± 0.57^a^	0.38 ± 0.16^d^	6.49 ± 0.23^c^
	TG52:6+O*	5.33 ± 0.16^a^	3.97 ± 0.32^b^	0.00 ± 00^d^	0.75 ± 0.05^c^
	TG54:2+O*	2.52 ± 0.20^b^	1.63 ± 0.60^c^	0.12 ± 0.01^d^	3.51 ± 0.20^a^
	TG54:8+2O*	1.34 ± 0.53^d^	5.05 ± 0.25^c^	37.92 ± 0.23^a^	19.63 ± 0.15^b^
	TG54:8+3O*	1.55 ± 0.41^b^	2.03 ± 0.50^a^	0.31 ± 0.01^c^	2.12 ± 0.04^a^
	TG54:6+Ox*	0.38 ± 0.20^d^	3.02 ± 0.13^c^	51.45 ± 0.29^a^	24.56 ± 0.41^b^

Summary of oxidized glycerolipids (nmol) in the roots of both soybean cultivars. Data represented are means ± standard errors for four sample replicates. Means in the same row accompanied by different superscripts represent significance differences (*) between the treatments, consisting of susceptible root control (ORC) and susceptible root inoculated (ORI); and tolerant root control (CRC) and tolerant root inoculated (CRI) from 10-day old plants. Significance level assessed at α = 0.05. The oxylipins detected were oxidized phosphatidylcholine (Ox-PC), oxidized phosphatidylethanolamine (Ox-PE), oxidized phosphatidic acid (Ox-PA), oxidized phosphatidylinositol (Ox-PI), and oxidized triacylglycerol (Ox-TG), O = monoxide, 2O = dioxide, 3O = trioxide and Ox = oxidized.

**Table 4:** Oxidized glycerolipids (nmol) in the stem of soybean cultivars following inoculation with *P. sojae*.

Lipid class	Oxidized glycerolipid	Relative abundance (nmol)			
		OSC	OSI	CSC	CSI
Phosphatidylcholine	PC36:6+2O*	1.85 ± 0.07^b^	0.19 ± 0.07^c^	0.35 ± 0.20^c^	3.75 ± 0.20^a^
	PC36:5+2O*	17.41 ± 0.40^b^	20.18 ± 0.58^a^	17.62 ± 0.66^b^	19.75 ± 0.16^a^
Phosphatidylethanolamine	PE38:6+O*	22.45 ± 0.31^b^	24.17 ± 0.60^a^	21.81 ± 0.35^b^	20.96 ± 0.36^c^
	PE38:6+2O*	18.07 ± 0.63^b^	20.18 ± 0.58^a^	17.63 ± 0.60^b^	19.53 ± 0.46^a^
Phosphatidic acid	PA34:3+O*	0.04 ± 0.00^c^	0.05 ± 0.02^c^	16.27 ± 0.21^a^	6.40 ± 0.17^b^
Triacylglycerol	TG54:8+2O*	1.76 ± 0.32^c^	0.69 ± 0.23^d^	9.79 ± 2.00^a^	6.20 ± 0.30^c^
	TG54:8+3O*	0.93 ± 0.02^c^	1.20 ± 0.90^b^	1.30 ± 0.30^b^	2.03 ± 0.90^a^
	TG52:6+O*	0.62 ± 0.13^c^	0.33 ± 0.21^c^	1.81 ± 0.20^b^	2.87 ± 0.85^a^
	TG54:9+O*	4.29 ± 1.55^a^	3.10 ± 0.72^b^	0.09 ± 0.05^d^	0.49 ± 0.14^c^
	TG60:9+5O*	4.24 ± 1.00^d^	6.37 ± 0.82^c^	22.46 ± 6.00^b^	24.22 ± 0.47^a^
	TG60:8+5O*	0.47 ± 0.20^d^	2.35 ± 1.16^c^	17.90 ± 5.00^b^	24.19 ± 0.62^a^
	TG60:10+6O*	47.05 ± 0.31^b^	67.13 ± 4.64^a^	0.17 ± 0.10^c^	0.00 ± 0.00^c^
	TG54:2+Ox*	0.00 ± 0.00^c^	0.00 ± 0.00^c^	5.26 ± 2.00^b^	7.34 ± 0.47^a^

Summary of oxidized glycerolipids (nmol) in the stems of both soybean cultivars. Data represented are means ± standard errors for four sample replicates. Means in the same row accompanied by different superscripts represent significance differences (*) between the treatments, consisting of susceptible stem control (OSC) and susceptible stem inoculated (OSI); and tolerant stem control (CSC) and tolerant stem inoculated (CSI) from 10-day old plants, at a significance level assessed at α < 0.05. The oxylipins detected were oxidized phosphatidylcholine (Ox-PC), oxidized phosphatidylethanolamine (Ox-PE), oxidized phosphatidic acid (Ox-PA), and oxidized triacylglycerol (Ox-TG), O = monoxide, 2O = dioxide, 3O = trioxide, 4O = tetroxide, 5O = pentoxide, 6O = hexoxide and Ox = oxidized.

Across all time points, as well as infected and non-infected plants, the relative abundance of the primary oxylipins in the root ranged between 0.00 to 58,117.16 nmol for the susceptible cultivar and 0.00 to 98,027.91 nmol for the tolerant cultivar (**Table 1**) while the relative abundance of the primary oxylipins in the stem ranged between 0.00 to 152,625.22 nmol for the susceptible cultivar and 0.00 to 147,879.60 nmol for the tolerant cultivar (**Table 2**). Similarly, the relative abundance of the oxidized glycerolipids in the root ranged between 0.38 ± 0.20 to 25.23 ± 0.45 nmol for the susceptible cultivar and 0.00 ± 00 to 51.45 ± 0.29 nmol for the tolerant cultivar (**Table 3**), and the relative abundance of the oxidized glycerolipids in the stem ranged between 0.00 ± 00 to 67.13 ± 4.46 nmol for the susceptible cultivar and 0.00 ± 00 to 24.22 ± 0.47 nmol for the tolerant cultivar (**Table 4**). Notably, the levels of seven primary oxylipins in the root and stem were significantly increased in the tolerant cultivar relative to the non-inoculated controls, but significantly reduced in susceptible cultivar in response to *P. sojae* infection and colonization. Contrarily, two primary oxylipins that were microbial origin, 12(S)-HpETE and 16,17-EpDPE were significantly increased in infected susceptible cultivar after 48 h, 72 h and 96 h post-infection but no changes were observed in non-infected control of both cultivars and infected tolerant cultivar after infection with the pathogen (**Tables 1**, **2**). The behavioral response differentiating primary oxylipins and oxidized glycerolipids in this study demonstrated that oxidized glycerolipids exhibit indirect physiological activities by acting as precursors for synthesis of primary oxylipins, while primary oxylipins exhibit direct strong antimicrobial activities to mitigate plant infection and colonization caused by pathogens.

### Phyto-oxylipin induction in susceptible and tolerant soybean cultivars in response to *P. sojae* infection

**Figure 5:**
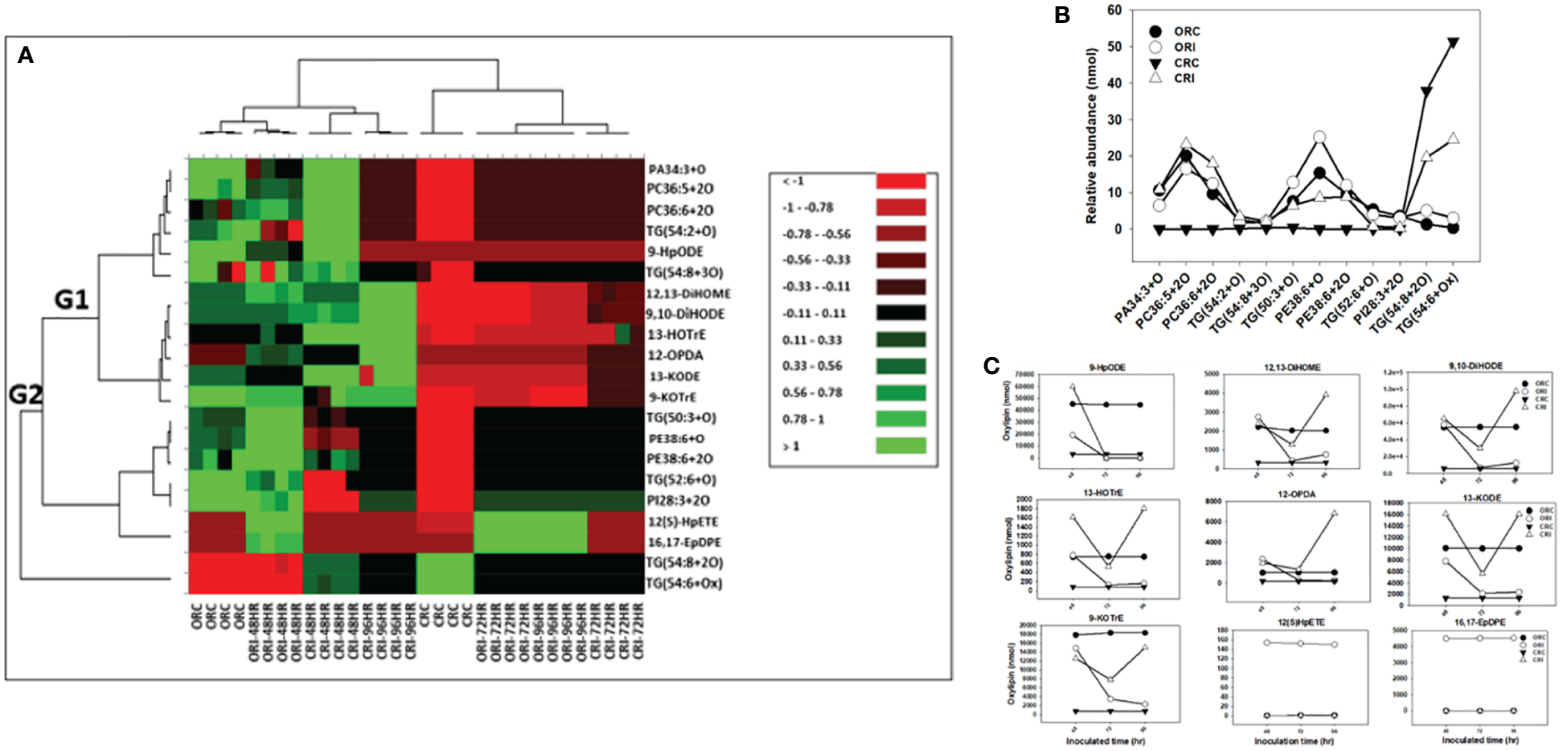
Changes in phyto-oxylipins biosynthesized in root of both soybean cultivars infected with *P. sojae* relative to non-infected plants. **(A)** Heat map showing clusters of oxylipins in susceptible (OX760-6) and tolerant (Conrad) inoculated or non-inoculated with *P. sojae.* Each soybean cultivar and treatment were clustered independently using ascendant order clustering established on Euclidian distance at 0.15 interquartile range. The left columns represent the cluster-separated root phyto-oxylipins, whereas the top columns separated the cultivars established on similarities in abundance of phyto-oxylipins. Red, black, and green colors denote lower, intermediate, and higher abundance of root phyto-oxylipins. Group 1 and 2 (G1 and G2) are induced phyto-oxylipins that were responsible cluster patterns in the heat map used to determine differences between susceptible and tolerant cultivars; **(B)** Line graphs showing significant changes in oxidized glycerolipids in the root of both cultivars following inoculation over 96 hours and **(C)** Line graphs showing significant changes in primary oxylipins in the root of both cultivars following inoculation for over 96 hours. The phyto-oxylipins observed to be significantly different in each group (G1 and G2) of the heat map are displayed in the line graphs. Values represents mean nanomole percent with n = 4 per time point. No detectable response was observed at 24 h of inoculation (data not shown).

**Figure 6:**
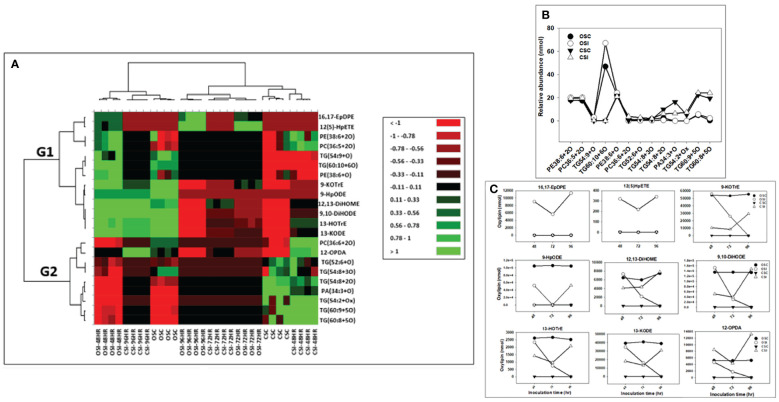
Changes in phyto-oxylipins biosynthesized in stem of both soybean cultivars infected with *P. sojae* relative to non-infected plants. **(A)** Heat map showing clusters of oxylipins in susceptible (OX760-6) and tolerant (Conrad) inoculated or non-inoculated with *P. sojae.* Each soybean cultivar and treatment were clustered independently using ascendant order clustering established on Euclidian distance at 0.15 interquartile range. The left columns represent the cluster separated stem phyto-oxylipins, whereas the top columns separated the cultivars established on similarities in abundance of phyto-oxylipins. Red, black, and green colors denote lower, intermediate, and higher abundance of stem phyto-oxylipins. Group 1 and 2 (G1 and G2) are induced oxylipins that were responsible cluster patterns in the heat map used to determine differences between susceptible and tolerant cultivars; **(B)** Line graphs showing significant changes in oxidized glycerolipids in the stem of both cultivars following inoculation over 96 hours and **(C)** Line graphs showing significant changes in primary oxylipins in the stem of both cultivars following inoculation for over 96 hours. The phyto-oxylipins observed to be significantly different in each group (G1 and G2) of the heat map are displayed in the line graphs. Values represents mean nanomole percent with n = 4 per time point. No detectable response was observed at 24 h of inoculation (data not shown).

Analysis of primary oxylipins demonstrated significant changes in the root and stem phyto-oxylipins between and within the two soybean cultivars during interaction with the oomycete *P. sojae.* **Figures 5A–C**, **6A–C** show the levels of oxylipin alterations that occurred during soybean-*P. sojae* interactions. The model quality (Q^2^) generated from PLS-DA explains 65% variability in the root and 60% variability in the stem. Heat maps (**Figures 5A**, **6A**) were prepared for both oxidized glycerolipids and primary oxylipins with important loadings representing the cultivar and treatment separation to further categorize the treatments based on the alterations observed in response to *P. sojae* infection. Meanwhile, no significant differences were observed between the time points for the control treated plants, therefore, averaged results were used in the heat map. The cut-off score for variables important in projection (VIP) results was defined as >1 (Ravipati et al., 2015; Nadeem et al., 2020). The 21 phyto-oxylipins in the root (12 oxidized glycerolipids and nine primary oxylipins) and 22 phyto-oxylipins in the stem (13 oxidized glycerolipids and nine primary oxylipins) were selected based on VIP results. The outcome from the heatmap demonstrated four distinct clusters of the soybean root and stem oxylipins following inoculation with *P. sojae* (**Figures 5A**, **6A**).

The heatmap ordinate root and stem phyto-oxylipins into two major groups (G), G1 and G2 (**Figures 5A**, **6A**). The susceptible and resistant cultivars could be distinguished at each time point and by inoculation status (**Figure 5A**). Differences were observed in phyto-oxylipins in both soybean cultivars, corresponding to G1, where the relative abundance of five oxidized glycerolipids, PA34:3+O, PC36:5+2O, PC36:6+2O TG(54:2+O), TG(54:8+3O), and seven primary oxylipins, 9-HpODE, 12,13-DiHOME, 9,10-DiHODE, 13-HOTrE, 12-OPDA, 13-KODE, and 9-KOTrE significantly increased in the tolerant cultivar at 48 h, 72 h and 96 h after infection with the pathogen, but contrarily, these oxylipins were significantly decreased in the susceptible cultivar at 48 h, 72 h and 96 h (**Figure 5A**). Though, 9-HpODE significantly increased when both the susceptible and the tolerant cultivars were challenged with *P. sojae* for 48 h, but were reduced at the 72 h and 96 h time points (**Figure 5A**). In G2, the relative abundance of seven oxidized glycerolipids, TG(50:3+O), PE38:6+O, PE38:6+2O, TG(52:6+O), PI28:3+3O, TG(54:8+2O) and TG(54:6+Ox significantly increased in the tolerant cultivar at 48 h, 72 h and 96 h after infection with the pathogen, but contrarily, these oxidized glycerolipids were significantly decreased in the susceptible cultivar at 48 h, 72 h and 96 h (**Figure 5A**). Two primary oxylipins, 12(S)-HpETE and 16,17-EpDPE that were microbial origin was significantly increased in infected susceptible cultivar after 48 h, 72 h and 96 h but no changes were observed in non-infected control of both cultivars and infected tolerant cultivar after infection with the pathogen. These results were supported by the relative abundance of the oxidized glycerolipids (**Figure 5B**) and the concentration of primary oxylipins (**Figure 5C**) in the root of both soybean cultivars. In a similar manner, the heat map groupings differentiated the stem oxylipins in the susceptible cultivar from the tolerant cultivar based on time of inoculation (**Figure 6A**). Differences were observed in phyto-oxylipins in both soybean cultivars, corresponding to G1, where the relative abundance of two primary oxylipins that were microbial origin, 12(S)-HpETE and 16,17-EpDPE were significantly increased in infected susceptible cultivar after 48 h, 72 h and 96 h post-infection but no changes were observed in non-infected control of both cultivars and infected tolerant cultivar after infection with the pathogen while seven primary oxylipins, 9-KOTrE, 9-HpODE, 12,13-DiHOME, 9,10-DiHODE, 13-HOTrE and 13-KODE, 12-OPDA were significantly increased in the tolerant cultivar but not in the susceptible cultivar at the 48 h, 72 h and 96 h after infection with *P. sojae.* Meanwhile, five oxidized glycerolipids (PE38:6+2O, PC36:5+2O, TG[54:9+O], TG[60:10+6O] and PE38:6+O) were generally significantly increased in the tolerant cultivar but not in the susceptible cultivar at the 72 h and 96 h time points, but their concentration were reduced in tolerant cultivars while increased in susceptible cultivar at 48 h after infection with *P. sojae* (**Figure 6A**). Oxylipins belonging to G2 consisted of only one primary oxylipin, 12-OPDA which was significantly increased in the tolerant cultivar but not in the susceptible cultivar at the 48 h, 72 h and 96 h after infection with *P. sojae.* Eight oxidized glycerolipids (PC36:6+2O, TG[52:6+O], TG[54:8+3O], TG[54:8+2O], PA34:3+O, TG[54:2+OX], TG[60:9+5O] and TG[60:8+5O]), we observed significant increases in these oxidized glycerolipids at all time points in the tolerant cultivar relative to the non-inoculated control while the same lipids were reduced in the susceptible cultivar at all three time points relative to the control in response to the pathogen infection (**Figure 6A**). These results were supported by the relative abundance of the oxidized glycerolipids (**Figure 6B**) and the concentration of primary oxylipins (**Figure 6C**) in the stem of both soybean cultivars. Generally, these results demonstrated that the levels of primary oxylipins in root and stem significantly increased in the tolerant soybean cultivar but were significantly reduced in the susceptible cultivar following inoculation with *P. sojae*.

### Spearman’s correlation between oxidized glycerolipids and primary oxylipins in soybean cultivars in response to *P. sojae* infection

**Figure 7:**
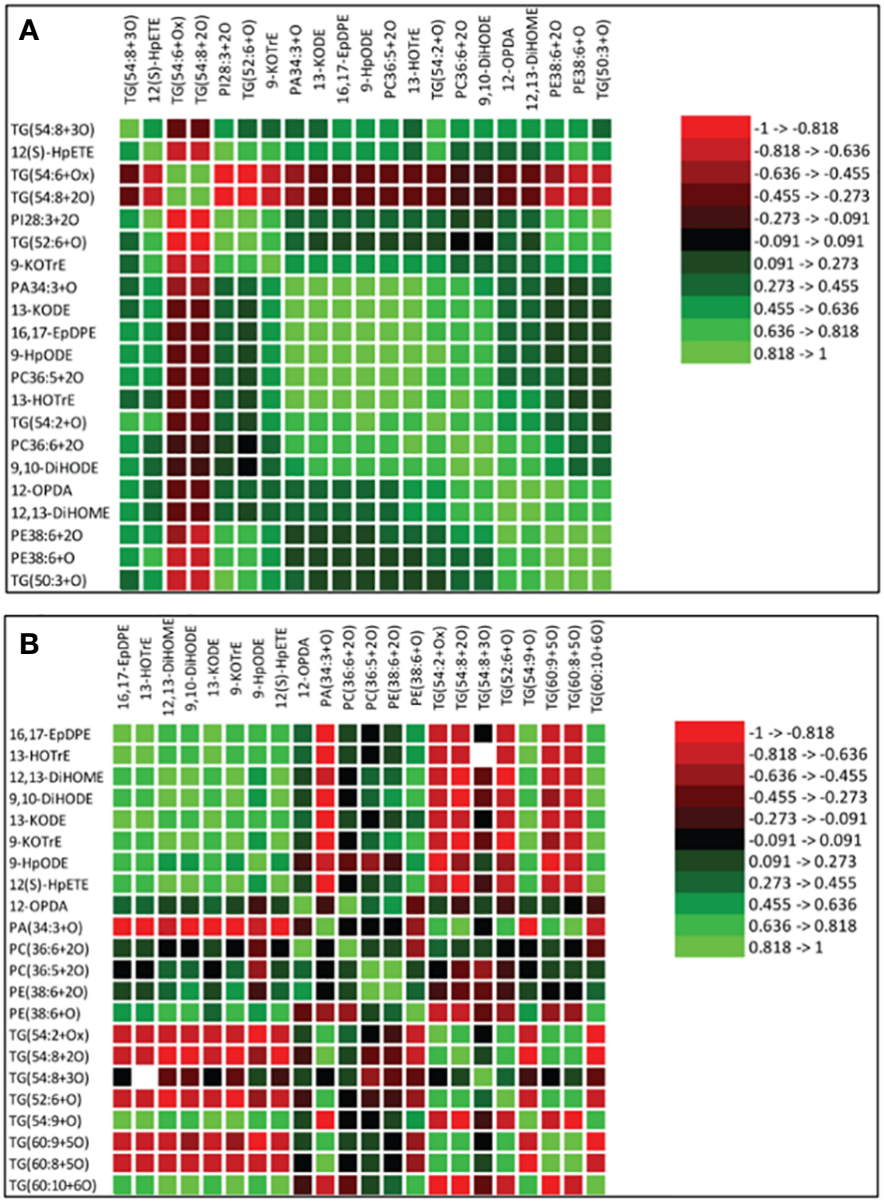
Spearman’s rank correlation coefficients heatmap between relative abundance of oxidized glycerolipids and primary oxylipins in the susceptible (OX760-6) and tolerant (Conrad) soybean cultivars. **(A)** Correlation between oxidized glycerolipids and primary oxylipins in the root of susceptible and tolerant soybean cultivars infected with pathogen relative to non-infected plants; **(B)** Correlation between oxidized glycerolipids and primary oxylipins in the stem of susceptible and tolerant soybean cultivars infected with pathogen relative to non-infected plants. Colors indicate the Spearman correlations’ ρ values, i.e. the correlation strength between oxidized glycerolipids and primary oxylipins. The ρ values between <-0.5 and > 0.5 have a significant value of P < 0.05. To generate the heatmap, cluster analyses were carried out using the group average method to cluster oxidized glycerolipids and primary oxylipins that have similar Spearman rank coefficients. 16,17-EpDPE = (4*Z*,7*Z*,10*Z*,13*Z*)-15-[3-[(*Z*)-pent-2-enyl] oxiran-2-yl] pentadeca-4,7,10,13-tetraenoic acid, 13-HOTrE = 10(E),12(Z),13S-hydroxy-9(Z),11(E),15(Z)-octadecatrienoic acid, 12,13-DiHOME = (*Z*)-12,13-dihydroxyoctadec-9-enoic acid, 9, 10-DiHODE = (12*Z*,15*Z*)-9,10-dihydroxyoctadeca-12,15-dienoic acid, 12-OPDA = 12-oxophytodienoic acid, 13-KODE = (9Z,11E)-13-Oxo-9,11-octadecadienoic acid, 9-KOTrE = 15(Z)-9-oxo-octadecatrienoic acid, 12(S)-HpETE = 12S-hydroperoxy-5(Z),8(Z),10(E),14(Z)-eicosatetraenoic acid, and 9-HpODE = 10(E*)*,12(E)-9-hydroperoxyoctadeca-10,12-dienoic acid, oxidized phosphatidylcholine (Ox-PC), oxidized phosphatidylethanolamine (Ox-PE), oxidized phosphatidic acid (Ox-PA), oxidized phosphatidylinositol (Ox-PI), and oxidized triacylglycerol (Ox-TG), O = monoxide, 2O = dioxide, 3O = trioxide and Ox = oxidized.

Spearman’s correlation coefficients of 12 oxidized glycerolipids and nine primary oxylipins are shown in **Figure 7A**. The correlation coefficient (r) was strongly positive for the relationships between 16,17-EpDPE, 13-HOTrE, 12,13-DiHOME, 9,10-DiHODE, 12-OPDA, 13-KODE, 9-KOTrE, 12(S)-HpETE, 9-HpODE and PA30:4+2O, PC36:5+2O, PC36:6+2O TG(54:2+O), TG(54:8+3O), TG(50:3+O), PE38:6+3O, PE38:6+2O, TG(52:2+O) and PI28:3+2O but strongly negative for TG(54:6+Ox) and TG(54:8+2O). The strongest significant positive correlation ranged between 0.455 to >1.000 for these relationships. Generally, strong positive correlations were observed between oxidized glycerolipids and primary oxylipins in the root of both soybean cultivars following inoculation with *P. sojae* infection (**Figure 7A**). Similarly, in the stems of both cultivars, strongly positive and negative correlations were observed in response to infection (**Figure 7B**). As demonstrated in **Figure 7B**, 12-OPDA was strongly positively correlated with oxidized glycerolipids with correlation ranging between 0.445 to > 0.818. The other primary oxylipins, 12,13-DiHOME, 9,10-DiHODE, 12(S)-HpETE, 9-KOTrE, 16,17-EpDPE, 13-KODE, 13-HOTrE, and 9-HpODE, exhibited significant positive correlation ranging between 0.273 to > 0.455 as well as significant inverse (negative) correlation ranging between -0.455 to > -0.273 with TG(60:8+5O), TG(60:9+5O) and TG(54:2+Ox).

### Phyto-oxylipins network analysis

**Figure 8:**
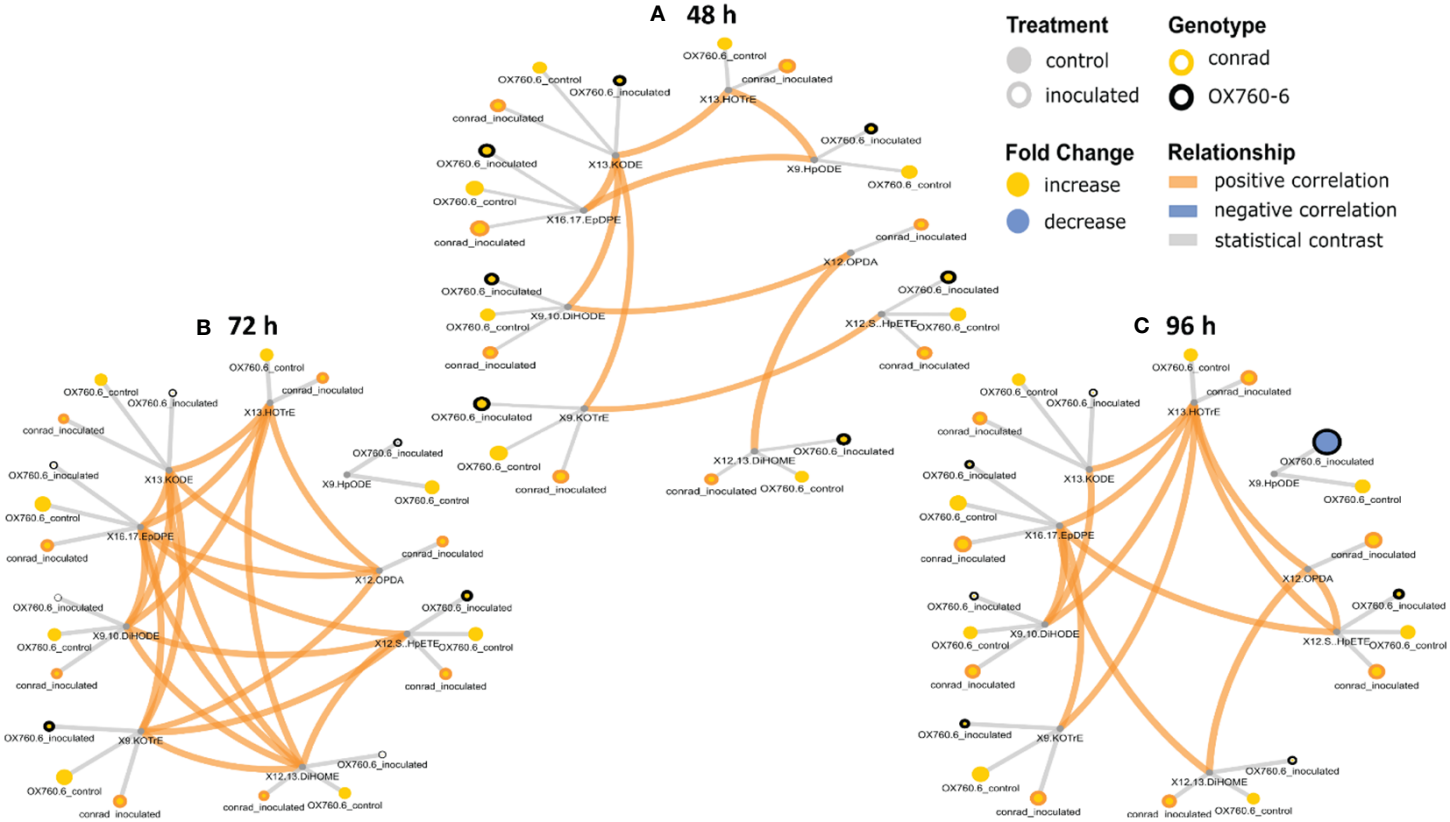
Oxylipin network displaying differences in root phyto-oxylipins in susceptible and resistant soybean cultivars following *P. sojae* inoculation relative to non-inoculated plants. **(A)** Control and inoculated susceptible soybean root (ORC and ORI), and control and inoculated tolerant soybean root (CRC and CRI) at 48 h time point, **(B)** control and inoculated susceptible soybean root (ORC and ORI), and control and inoculated tolerant soybean root (CRC and CRI) at 72 h time point, **(C)** control and inoculated susceptible soybean root (ORC and ORI), and control and inoculated tolerant soybean root (CRC and CRI) at 96 h time point. The network of phyto-oxylipins demonstrate fold changes in nine root primary oxylipins following infection with *P. sojae*. Lipid SMILES identifiers were applied to determine PubChem molecular fingerprints and phyto-oxylipin similarity structure. Mapped structural networks showing significance of fold changes in phyto-oxylipins were calculated for all comparisons. The network visualizes the phyto-oxylipins with connections established on structural Tanimoto similarity ≥ 0.8 (edge width: 0.8-1.0). Node size shows fold changes of means between comparisons and color demonstrates the direction of alteration compared to control (yellow: increased; blue: decreased; gray: statistical contrast). Node shape shows phyto-oxylipin structural type (gray circle: control, rounded gray: inoculated; rounded yellow: Conrad (tolerant soybean cultivar) and rounded black: OX760-6 (susceptible soybean cultivar), brown = positive correlation and blue = negative correlation. Oxylipins displaying significant differences between treatment groups (*p-value* ≤ 0.05) are denoted with rounded yellow.

**Figure 9:**
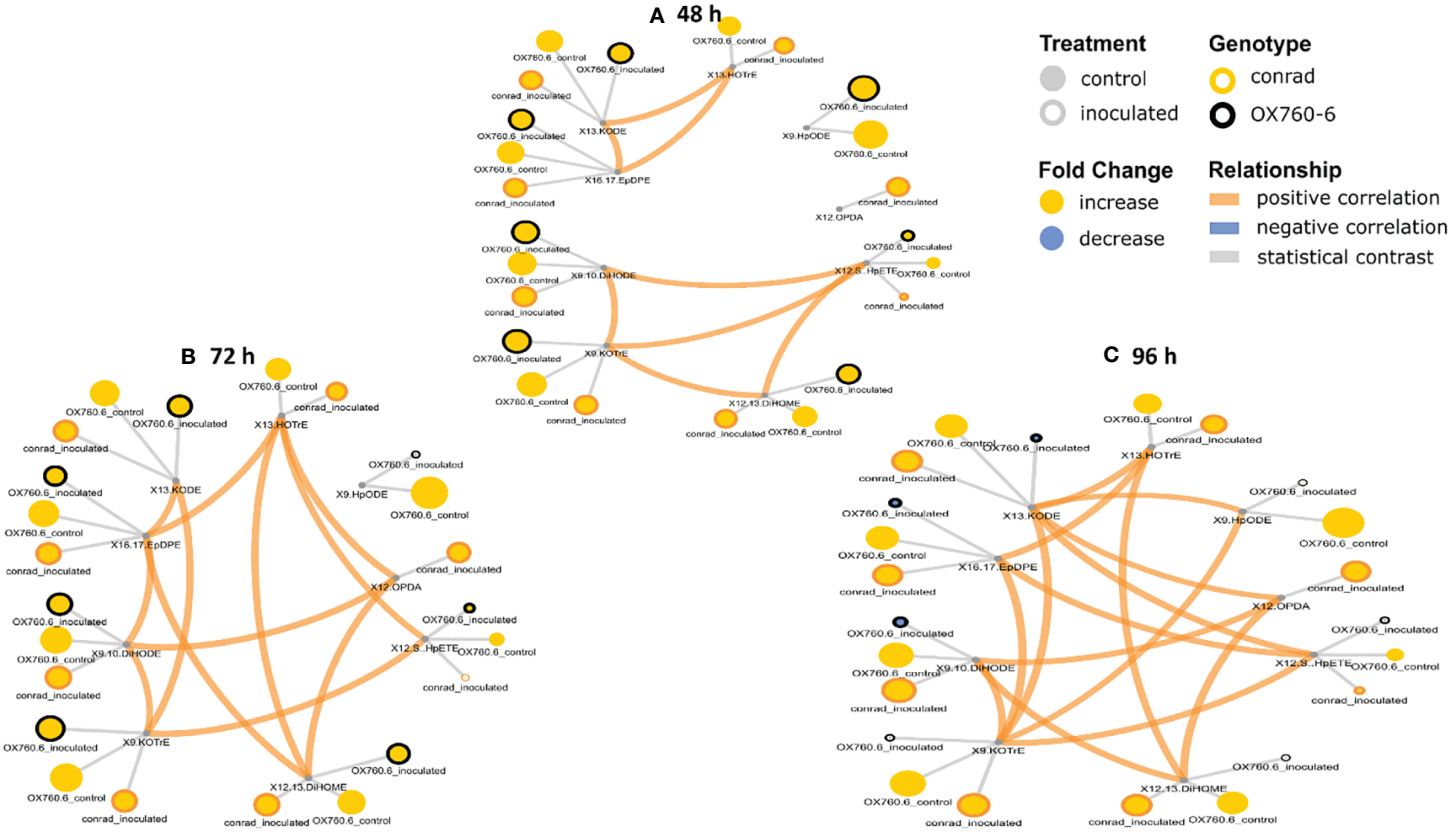
Oxylipin network displaying differences in stem phyto-oxylipins in susceptible and resistant soybean cultivars following *P. sojae* inoculation relative to non-inoculated plants. **(A)** Control and inoculated susceptible soybean stem (OSC and OSI), and control and inoculated tolerant soybean stem (CSC and CSI) at 48 h time point, **(B)**control and inoculated susceptible soybean stem (OSC and OSI), and control and inoculated tolerant soybean stem (CSC and CSI) at 72 h time point, and **(C)** control and inoculated susceptible soybean stem (OSC and OSI) and control and inoculated tolerant soybean stem (CSC and CSI) at 96 h time point. The network of phyto-oxylipins demonstrates fold changes in nine stem primary oxylipins following infection with *P. sojae.* Lipid SMILES identifiers were applied to determine PubChem molecular fingerprints and phyto-oxylipin similarity structure. Mapped structural networks, showing significance of fold changes in phyto-oxylipins were calculated for all comparisons. The network visualizes the phyto-oxylipins with connections established on structural Tanimoto similarity ≥ 0.8 (edge width: 0.8-1.0). Node size shows fold changes of means between comparisons and color demonstrates the direction of alteration compared to control (yellow: increased; blue: decreased; gray: statistical contrast). Node shape shows phyto-oxylipin structural type (gray circle: control, rounded gray: inoculated; rounded yellow: Conrad (tolerant soybean cultivar) and rounded black: OX760-6 (susceptible soybean cultivar), brown = positive correlation and blue = negative correlation. Oxylipins displaying significant differences between treatment groups (*p-value* ≤ 0.05) are denoted with rounded yellow.

It was demonstrated that phyto-oxylipins significantly increase among the primary oxylipins at all time points in the tolerant cultivar relative to the non-inoculated control, while the same lipids were reduced in the susceptible cultivar at all three time points relative to the control in response to the pathogen infection. Selected root and stem oxylipins among the inoculated susceptible cultivar showed significant decreases compared to the reference group at 96 h (**Figures 8**, **9**). Generally, almost all the primary oxylipins in the root, including 12-OPDA, 13-HOTrE, 12,13-DiHOME, 13-KODE, 9-KOTrE, 9-HpODE and 9, 10-DiHODE, were significantly decreased in the susceptible cultivar at 48 h, 72 h and 96 h following inoculation with *P. sojae* relative to the non-inoculated control, except 16,17-EpDPE and 12(S)-HpETE which was increased after inoculated at 48 h, 72 h and 96 h. Contrary to susceptible cultivar, seven of the primary oxylipins in the root of the tolerant cultivar, which were 13-HOTrE, 12,13-DiHOME, 13-KODE, 9-KOTrE, 12-OPDA, 10-DiHODE and 9, 9-HpODE were significantly increased at all time points following inoculation relative to the non-inoculated control; the two exception were 16,17-EpDPE and 12(S)-HpETE which significantly decreased after 48 h, 72 h and 96 h inoculation (**Figure 8**). Similarly, almost all the primary oxylipins in stem, including 13-HOTrE, 12-OPDA, 12,13-DiHOME, 13-KODE, 9-KOTrE, 9-HpODE and 9, 10-DiHODE, were significantly decreased in the susceptible soybean cultivar at all time points following inoculation relative to the non-inoculated control, except 16,17-EpDPE and 12(S)-HpETE which increased after 48 h, 72 h and 96 h inoculation. Contrary to the susceptible cultivar, seven of the primary oxylipins in stem, which included 13-HOTrE, 12,13-DiHOME, 13-KODE, 9-KOTrE, 12-OPDA, 9-HpODE, and 9, 10-DiHODE were significantly increased in the tolerant cultivar at all time points following inoculation relative to the non-inoculated control, except 16,17-EpDPE and 12(S)-HpETE which decreased after 48 h, 72 h and 96 h inoculation. Notably, there were fold increase in 9-HpODE, 13HOTrE, 13-KODE, 9,10-DiHODE, 9-KOTrE and 12,13-DiHOME in the root and stem control but decreased in the infected root and stem of susceptible cultivar at 48 h, 72 h and 96 h time points. In contrast, fold increase of these oxylipins were observed in the infected root and stem but decreased in control root and stem of tolerant cultivar at 48 h, 72 h and 96 h time points (**Figures 8**, **9**). Similarly, 12-OPDA was found to be decreased both in the control and infected root and stem of susceptible cultivar at 48 h, 72 h and 96 h time points but fold increase were observed in the infected root and stem of tolerant cultivar at 48 h, 72 h and 96 h time points (**Figures 8**, **9**).

## Discussion

Plant-pathogen interaction is a complicated process, induced by the pathogen- and plant-derived molecules which majorly involve sugars, lipids, proteins and lipopolysaccharides (Gupta et al., 2015; Abdul Malik et al., 2020). Secreted molecules, obtained from the pathogens, are the main factors which regulate their pathogenicity and permit successful host colonization and infection (Gupta et al., 2015). These plant derived molecules are implicated in the recognition of pathogens in order to activate the defense response (Adigun et al., 2020). The initial interaction between the plants and pathogens take place in the apoplast and is induced by the recognition of pathogen elicitors through plant’s receptor proteins (Gupta et al., 2015; Abdul Malik et al., 2020). These pathogen elicitors are known as pathogen-associated molecular patterns and are recognized by the membrane-localized pattern recognition receptors of plants (Abdul Malik et al., 2020). These membrane localized pattern recognition receptors are particularly susceptible to alteration in membrane lipidome, and plant immunity mediated by fatty acids such as oxylipins. Generally, pathogen appears to overtake the immune system of susceptible soybean by modulating their cell surfaces by releasing proteins to degrade plant immunity conferring cell structures (e.g. reinforced epidermal walls) as well as the pathogen producing its own oxylipins mediate signal induction factors that adversely modulated the plant immune response cascade resulting in catabolism of endogenous morphological structures such as biogenic crystals post infection limiting the plant ability to circumvent colonization and infection by the pathogen (Vailleau et al., 2007).

Phytophthora root and stem-rot is one of the major destructive soybean diseases and the causal agent is oomycete *P. sojae* resulting in global annual crop losses of 10-20 $US (Wrather et al., 2010; Chi et al., 2021; Giachero et al., 2022). Environmentally sustainable agricultural practices are now embraced to prevent phytopathogen attack in plants and to enhance plant health. Higher plants possess sophisticated strategies by which to defend against stresses from infectious pathogens. Some defense mechanisms used by higher plants include remodulation of membrane lipidome (Adigun et al., 2021) and production of bioactive compounds has been shown to be effective response strategies to limit pathogen infection (Thomas et al., 2007; Adigun et al., 2020). For instance, biosynthesis of oxygenated PUFAs generally called oxylipins, is one of the early mechanisms of plant’s defense responses against pathogenic bacterial and fungal infection (Blée, 2002; Howe and Schilmiller, 2002).

Based on the limited understanding of the biochemical and physiological properties of oxylipins, a comprehensive study of oxylipins was generated from the root and stem of tolerant and susceptible soybean cultivars challenged with *P. sojae* to examine the alterations in oxylipin levels across three time points. All identified primary oxylipins generated from both soybean cultivars demonstrated significant alterations in response to infection, and oxidized glycerolipids generated from membrane lipids following oxidation of PUFAs. These PUFAs are predicted to serve as substrates for the biosynthesis of primary oxylipins following *P. sojae* infection. At 48 h, 72 h and 96 h post infection by *P. sojae*, compare to controls, we observed that oxylipins significantly decreased in the root and stem of susceptible soybean while they significantly increased in the root and stem of tolerant soybean. The results obtained from our studies are in agreement with the responses observed for different classes of oxylipins reported in literature. For instance, studies have shown that application of synthetic JA to tomato foliage triggers a systemic effect that allows the plant to resist root-knot nematode invasion (Cooper et al., 2005). This was accompanied by the production of JA to enclose and limit nematode invasion at to the initial area of infection, thereby inhibit nematode infection (Poveda et al., 2020). Also, other studies have demonstrated the effects of JA-induced defense responses on pathogenic organisms and the use of exogenous MeJA was observed to induce systemic resistance in the root of oats and spinach against pathogenic nematodes (Soriano et al., 2004). It has been well established that jasmonates play active roles during foliar and root defense responses to infection (Smith et al., 2009).

Similarly, several studies have implicated LOXs and their derivatives in the plant defense response against diverse pathogens (Kolomiets et al., 2001). For instance, a novel cyclopentenone, 10-oxo-11-phytodienoic acid synthesized through 9-LOX activities, and which is an isomer of 12-OPDA, the precursor of jasmonate, was isolated from young tubers and stolons of potato (*Solanum tuberosum*). It is possible that 9-LOX may play a role during jasmonate biosynthesis to control tuber growth and also function in the defense response against pathogenic attack (Kolomiets et al., 2001). Moreover, the application of some hydroperoxide derivatives of oxylipins have been demonstrated to inhibit conidial germination and elongation of germ-tube of the rice blast pathogen *Pyricularia oryzae* (Naor et al., 2018), and C18:2 hydroperoxides have demonstrated toxic effects on *Saccharomyces cerevisiae* (Naor et al., 2018). Furthermore, it was demonstrated that following pathogenic attack of the moss *Physcomitrella patens* by various microbial pathogens including *Pectobacterium carotovorum, Pectobacterium wasabiae* and *Botrytis cinerea*, the host induces a defense response by elevating the levels of endogenous FFAs and activating gene transcription encoding various LOXs, AOS, and OPDA acid reductase (Ponce De León et al., 2007; Ponce De León et al., 2012). In these pathosystems, the 13-/12-LOX pathways were suggested to be activated after pathogen attack. The transcript levels of PpLOX1 and PpLOX6 were increased following infection by *Pythium cinerea* and *Botrytis cinerea* respectively, and the concentrations of OPDA increased in response to both pathogens (Oliver et al., 2009; Ponce De León et al., 2012). In the present study, we found that primary oxylipins 9-HpODE, 12,13-DiHOME, 9,10-DiHODE, 12-OPDA, 9-KOTrE, 13-HOTrE, and 13-KODE were significantly decreased in the susceptible soybean cultivar in contrast to the significant increase observed in the tolerant soybean cultivar. These subclasses of oxylipins mediated tolerance to *P. sojae* infection in tolerant soybean as a function of time. Contrarily, 16,17-EpDPE and 12(S)-HpETE were observed to be significantly increased in the root and stem of the susceptible soybean cultivar, and they were known to be microbial-derived oxylipins associated with pathogenesis in susceptible soybean cultivar (Niu and Keller, 2019). Taken together, these results demonstrate that oxylipins participate in early defenses in soybean response to *P. sojae* infection.

The oxidized glycerolipid network analysis presents a significant challenge due to a lack of well-defined biochemical interaction databases and general lipid enzyme substrate promiscuity among FFA, membrane and neutral lipids. When lipid structures are known, estimates of similarities among lipid activities can be inferred based on structural similarities or mass spectra. Regularized correlations between lipid measurements were used to calculate primary oxylipin interaction networks for each of the three inoculation time points. All observed conditionally independent correlations between lipids were positive which can occur in cases where lipids are in homeostasis or share storage and sources of release (Demarsay, 2005). Lipid statistical contrasts between groups can be compared between time points to identify patterns of change. The network demonstrates the connectivity between the changes in phyto-oxylipins induction and the oxylipin biosynthesis pathway in the tolerant cultivar as defense response to *P. sojae* invasion. Generally, there is scarcity of information on the function of oxylipin induction to determine either compatible or incompatible interactions governing tolerance or susceptibility in the soybean-*P.sojae* pathosystem. The unique level of alterations in oxylipin induction between susceptible and tolerant cultivars showed that primary oxylipins 9-HpODE, 12,13-DiHOME, 9,10-DiHODE, 12-OPDA, 9-KOTrE, 13-HOTrE, and 13-KODE were significantly decreased in the root and stem of the susceptible soybean cultivar in contrast to the significant increase observed in the root and stem of the tolerant soybean cultivar (**Figures 5**, **6**). Studies have demonstrated that these oxylipins play active roles during plant disease resistance, or are involved in plant defense strategies against pathogen invasion (Blée, 2002).

**Figure 10:**
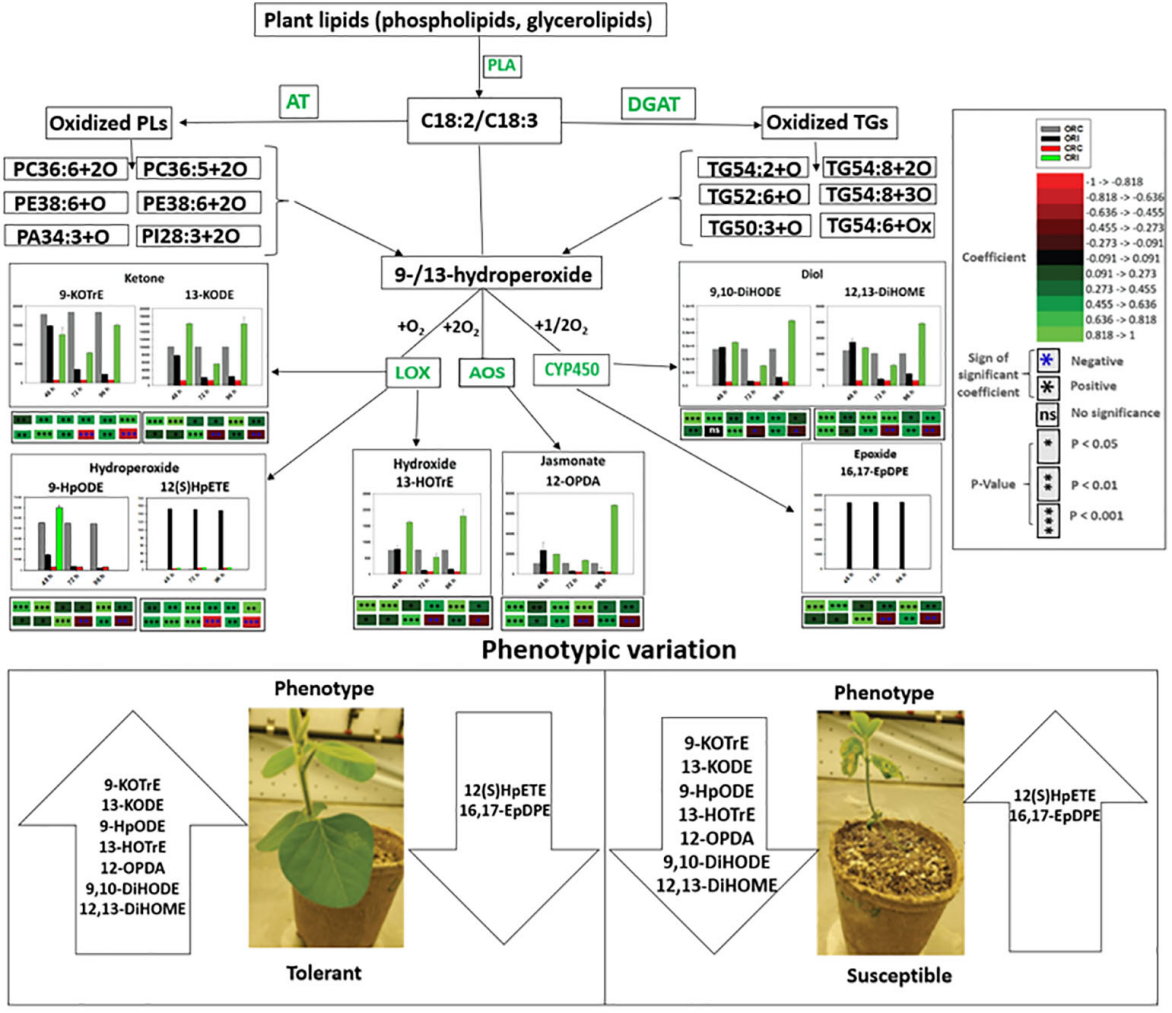
Proposed pathways demonstrating the mechanisms that may be connected with oxidized glycerolipid and primary oxylipin biosynthesis, and disease susceptibility or tolerance in both tolerant (OX760-6) and resistant (Conrad) soybean cultivars at 48 h, 72 h and 96 h after challenge with *P. sojae*. The most significantly altered phyto-oxylipins biosynthesized in root of susceptible and tolerant soybean cultivars following inoculation with *P. sojae* are presented in this diagram. We propose that following infection with *P. sojae*, polyunsaturated fatty acids (C18:3 and C18:2) from membrane complex lipids were hydrolyzed by phospholipase A followed by oxidation involving one, two or four oxygen atoms to synthesize oxidized glycerolipids. Acyltransferases biosynthesize oxidized phospholipids, and diacylglycerol acyltransferases biosynthesize oxidized triacylglycerol. These oxidized lipids appear to serve as potential precursor for the primary oxylipins forming the hydroperoxides based on the strong correlations between these oxidized glycerolipids and primary oxylipins. These hydroperoxides are further metabolized through enzymatic activities to produce various array of oxylipins catalyzed by LOX, CYP450 and AOS. The strongest correlations were observed between the following glycerolipids: PC36:6+2O, PC36:5+2O, PE38:6+O, PE38:6+2O, PA34:3+O, PI28:3+2O, TG50:3+O, TG52:6+O, TG54:2+O and TG54:8+3O and primary oxylipins, 9-HpODE, 12,13-DiHOME, 9,10-DiHODE, 12-OPDA, 9-KOTrE, 13-HOTrE and 13-KODE. The tolerant cultivar appears to produce several folds higher level of select oxylipins (jasmonates, diols, epoxides, hydroperoxides, ketones and hydroxides) in response to infection beginning at 48 h after inoculation over a 96 h time point. In contrast, these oxylipins are induced at lower levels in the susceptible soybean cultivars except 12(S)-HpETE and 16,17-EpDPE that appears to produce several folds higher level in infected susceptible cultivar. The levels of primary oxylipins produced in the root of tolerant cultivar were generally increased across the time points (48 h, 72 h and 96 h) but reduced in the root of susceptible cultivar and may be associated with the successful strategy used by tolerant soybean cultivar to limit *P. sojae* infection. These properties appear to be demonstrated from the phenotypic variation of both tolerant and susceptible soybean cultivars. AOS = allene oxide synthase, and CYP450 =cytochrome P450. 9-KOTrE = 15(Z)-9-oxo-octadecatrienoic acid, 13-KODE = (9Z,11E)-13-Oxo-9,11-octadecadienoic acid, 9-HpODE = 10(E*)*,12(E)-9-hydroperoxyoctadeca-10,12-dienoic acid, 12(S)-HpETE = 12S-hydroperoxy-5(Z),8(Z),10(E),14(Z)-eicosatetraenoic acid, 13-HOTrE = 10(E),12(Z),13S-hydroxy-9(Z),11(E),15(Z)-octadecatrienoic acid, 12-OPDA = 12-oxophytodienoic acid, (9Z,11E)-13-Oxo-9,11-octadecadienoic acid (13-KODE), 16,17-EpDPE = (4*Z*,7*Z*,10*Z*,13*Z*)-15-[3-[(*Z*)-pent-2-enyl] oxiran-2-yl] pentadeca-4,7,10,13-tetraenoic acid, 9, 10-DiHODE = (12*Z*,15*Z*)-9,10-dihydroxyoctadeca-12,15-dienoic acid, and 12,13-DiHOME = (*Z*)-12,13-dihydroxyoctadec-9-enoic acid. The phenotypes of two soybean cultivars infected by *P. sojae* at all examined time points is shown in **Figure S4**. Control susceptible soybean root (ORC), inoculated susceptible soybean root (ORI), control tolerant soybean root (CRC), inoculated tolerant soybean root (CRI). Soybean roots and stems demonstrated the same variability in quantitative response levels in both tolerant and susceptible cultivars when challenged with pathogen infection.

**Figure 11:**
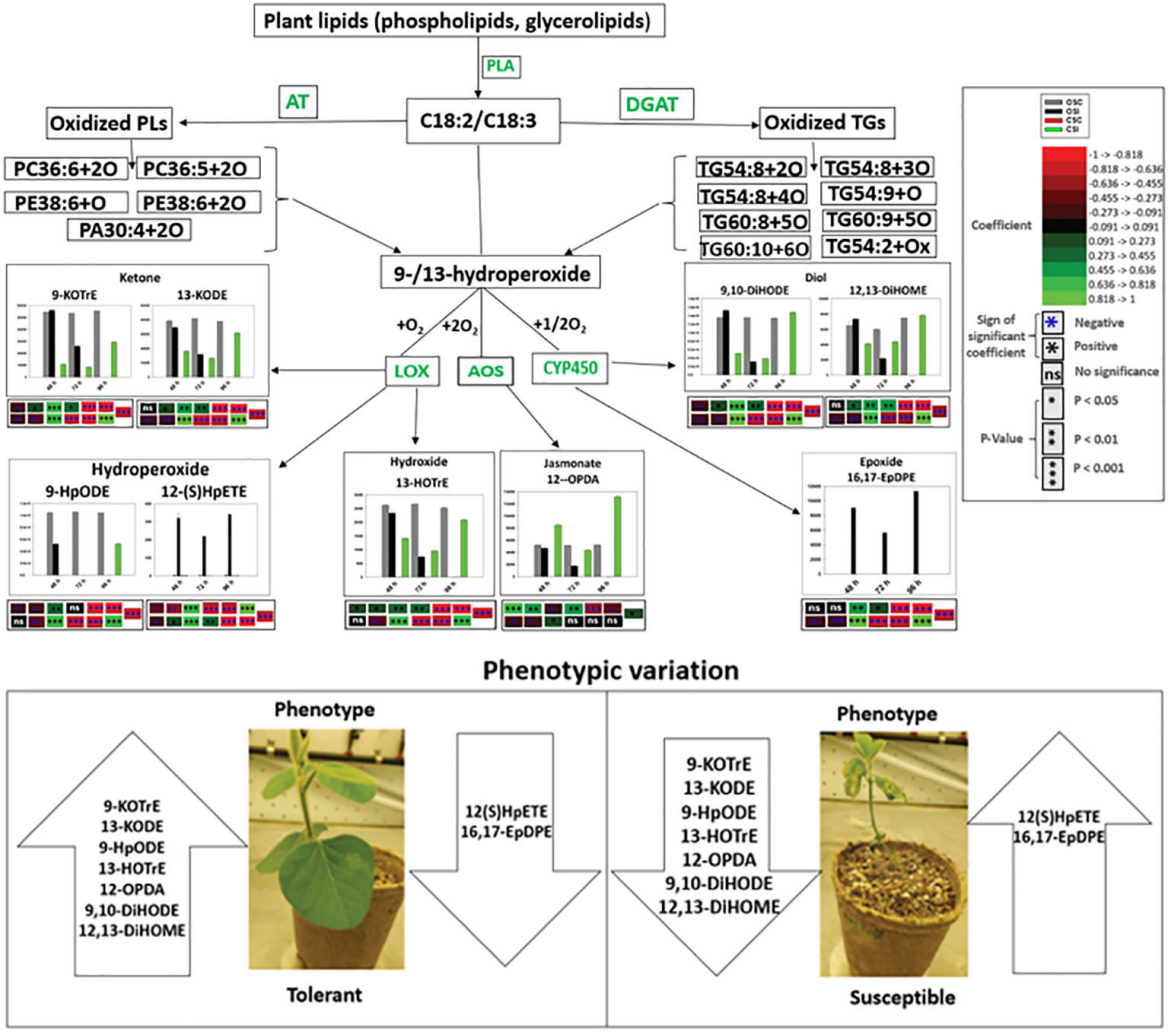
Proposed pathways demonstrating the mechanisms that may be connected with oxidized glycerolipid and primary oxylipin biosynthesis, and disease susceptibility or tolerance in both tolerant (OX760-6) and resistant (Conrad) soybean cultivars at 48 h, 72 h and 96 h after challenge with *P. sojae*. The most significantly altered phyto-oxylipins biosynthesized in stem of susceptible and tolerant soybean cultivars following inoculation with *P. sojae* are presented in this diagram. We propose that following infection with *P. sojae*, polyunsaturated fatty acids (C18:3 and C18:2) from membrane complex lipids were hydrolyzed by phospholipase A followed by oxidation involving one, two or four oxygen atoms to synthesize oxidized glycerolipids. Acyltransferases biosynthesize oxidized phospholipids, and diacylglycerol acyltransferases biosynthesize oxidized triacylglycerol. These oxidized lipids appear to serve as potential precursor for the primary oxylipins forming the hydroperoxides based on the strong correlations between these oxidized glycerolipids and primary oxylipins. These hydroperoxides are further metabolized through enzymatic activities to produce various array of oxylipins catalyzed by LOX, CYP450 and AOS. The strongest correlations were observed between the following glycerolipids: PC36:6+2O, PC36:5+2O, PE38:6+O, PE38:6+2O, TG54:8+3O, TG54:9+O, TG60:9+5O, TG60:10+6O and TG54:2+Ox and primary oxylipins, 9HpODE, 12,13-DiHOME, 9,10-DiHODE, 12-OPDA, 9-KOTrE, 13-HOTrE and 13-KODE. The tolerant cultivar appears to produce several folds higher level of select oxylipins (jasmonates, diols, epoxides, hydroperoxides, ketones and hydroxides) in response to infection beginning at 48 h after inoculation over a 96 h time point. In contrast, these oxylipins are induced at lower levels in the susceptible soybean cultivars except 12(S)-HpETE and 16,17-EpDPE that appears to produce several folds higher level in infected susceptible cultivar. The levels of primary oxylipins produced in the stem of tolerant cultivar were generally increased across the time points (48 h, 72 h and 96 h) but reduced in the stem of susceptible cultivar and may be associated with the successful strategy used by tolerant soybean cultivar to limit *P. sojae* infection. These properties appear to be demonstrated from the phenotypic variation of both tolerant and susceptible soybean cultivars. AOS = allene oxide synthase, and CYP450 =cytochrome P450. 9-KOTrE = 15(Z)-9-oxo-octadecatrienoic acid, 13-KODE = (9Z,11E)-13-Oxo-9,11-octadecadienoic acid, 9-HpODE = 10(E*)*,12(E)-9-hydroperoxyoctadeca-10,12-dienoic acid, 12(S)-HpETE = 12S-hydroperoxy-5(Z),8(Z),10(E),14(Z)-eicosatetraenoic acid, 13-HOTrE = 10(E),12(Z),13S-hydroxy-9(Z),11(E),15(Z)-octadecatrienoic acid, 12-OPDA = 12-oxophytodienoic acid, (9Z,11E)-13-Oxo-9,11-octadecadienoic acid (13-KODE), 16,17-EpDPE = (4*Z*,7*Z*,10*Z*,13*Z*)-15-[3-[(*Z*)-pent-2-enyl] oxiran-2-yl] pentadeca-4,7,10,13-tetraenoic acid, 9, 10-DiHODE = (12*Z*,15*Z*)-9,10-dihydroxyoctadeca-12,15-dienoic acid, and 12,13-DiHOME = (*Z*)-12,13-dihydroxyoctadec-9-enoic acid. The phenotypes of two soybean cultivars infected by *P. sojae* at all examined time points is shown in **Figure S4**. Control susceptible soybean stem (OSC), inoculated susceptible soybean stem (OSI), control tolerant soybean stem (CSC), inoculated tolerant soybean stem (CSI). Soybean roots and stems demonstrated the same variability in quantitative response levels in both tolerant and susceptible cultivars when challenged with pathogen infection.

Biosynthesis of oxylipins has been characterized in other systems (Genva et al., 2019). Polyunsaturated fatty acids such as C18:3 and C18:2 may be hydrolyzed by one, two or four oxygen atoms through phospholipase A and undergo further enzymatic reactions to generate oxidized glycerolipids (Blée, 2002; Liu et al., 2019). Acyltransferases biosynthesize oxidized phospholipids (Domingues et al., 2008; Liu et al., 2019), and diacylglycerol acyltransferases biosynthesize oxidized triacylglycerol (Irshad et al., 2017). These oxidized glycerolipids could act as potential precursors of primary oxylipins by further conversions of hydroperoxides through the activities of LOX, AOS and CYP450 to produce phyto-oxylipins in soybean tissues (**Figures 7**, **10**, **11**). In the root and stems of both soybean cultivars, the strongest correlations were observed between glycerolipids and primary oxylipins (**Figures 7**, **10**, **11**). The levels of primary oxylipins produced in the tolerant cultivar were generally increased across the time points (48 h, 72 h and 96 h) but reduced in the susceptible cultivar, and it seems that it was signally pathway activated that may appear to be the route tolerance or protection achieved in tolerant cultivar (**Figures 10**, **11**). Various groups of enzymes have been shown to participate in oxylipin formation, and radical pathways are also important (Blée, 2002). These enzymes, including LOX and α-dioxygenase, insert atoms of oxygen into FA chains and initiate pathways involving specialized cytochrome P450 monooxygenases that may be responsible for their downstream regulation (Blée, 2002; Griffiths, 2015). Furthermore, enzymes like AOS leading to JA signaling which may be responsible for synthesizing pathogen defensive volatiles, as well as POs and DESs that involved in producing antimicrobials may form part of the biochemical mediated response mounted by tolerant or resistant plants to limit pathogen infection (Prost et al., 2005; Griffiths, 2015). These oxylipin species could serve as biomarkers for disease susceptibility or tolerance by soybean cultivars when infected by pathogens (**Figures 10**, **11**). Based on our knowledge, the study of phyto-oxylipins and their rapid induction in the root and stem of susceptible and tolerant soybean cultivars in response to *P. sojae* infection, has not been previously reported in the literature. However, further study needs to be done to assess the gene expression levels associated with the pathway activated to further validate the proposed mechanism.

In conclusion, the results we present in this study demonstrated how phyto-oxylipin mediated plant immunity been regulated differently in both tolerant and susceptible soybean cultivars in the response to *P. sojae* infection. Knowledge of phyto-oxylipins in lipid mediate plant immunity has recently increased interest in the roles of phyto-oxylipin anabolism in plant defense responses against various infectious pathogens. Based on this, the assumed roles of phyto-oxylipins include activation of defense gene expression in plants, participation in plant defense by functioning as signaling molecules, directly serving as antimicrobial compounds, and regulating PCD. The exact contribution of these phyto-oxylipins in soybean defense against pathogen infection remains unknown. However, this study has shown unequivocally that most unique oxylipins including 9-HpODE, 12,13-DiHOME, 9,10-DiHODE, 12-OPDA, 9-KOTrE, 13-HOTrE, and 13-KODE were significantly upregulated after inoculation in tolerant soybean cultivar but downregulated in susceptible soybean cultivar, suggesting that these molecules may be a critical component of the defense strategies used in tolerant cultivar against *P. sojae* infection. In contrast, oxylipins that are microbial origin, 16,17-EpDPE and 12(S)-HpETE were upregulated in infected susceptible cultivar but downregulated in tolerant cultivar. In addition, the intact oxidized lipids that act as precursors for biosynthesis of primary oxylipins generally followed the same pattern as primary oxylipins in both soybean when challenged with *P. sojae* infection. Therefore, further study is required to better understand the role of both intact oxidized lipids and primary oxylipins as components of disease tolerance in tolerant soybean cultivars. Thus, genomic analysis and enzyme assays associated with both intact oxidized lipids and primary oxylipin biosynthesis may reveal the classes of genes distinctly upregulating the oxylipin mediated plant immunity in tolerant soybean cultivar during pathogen infection. This may identify targeted oxylipin biomarkers that are correlated with upregulated genes; therefore, the targeted genes could be a potential application to be used by breeders in pyramiding resistant soybean genotypes that could enhance resistance or higher tolerance to *P. sojae.* Therefore, this study has revealed novel evidence of various phyto-oxylipins mediated plant immunity and potential enzymatic pathways in soybean that may be leveraged to improve global soybean’s production as a strategy to reduce the over 10-20B USD in soybean crop losses.

## Data availability statement

The original contributions presented in the study are included in the article/**Supplementary Material**. Further inquiries can be directed to the corresponding authors.

## Author contributions

OA performed laboratory analysis. OA, TP, DG, and MN worked on data analysis. OA drafted the manuscript. RT, LJ, MC, and LG planned and designed the research. All authors contributed to the article and approved the submitted version.
