# Supplementary material for: Phyto-oxylipin mediated plant immune response to colonization and infection in the soybean-Phytophthora sojae pathosystem
Source: Front Plant Sci. 2023 May 12;14:1141823. doi: 10.3389/fpls.2023.1141823 (PMC10219219; doi:10.3389/fpls.2023.1141823)
Supplement: Supplementary file 1 [file DataSheet_1.docx]

**Supplemental materials**


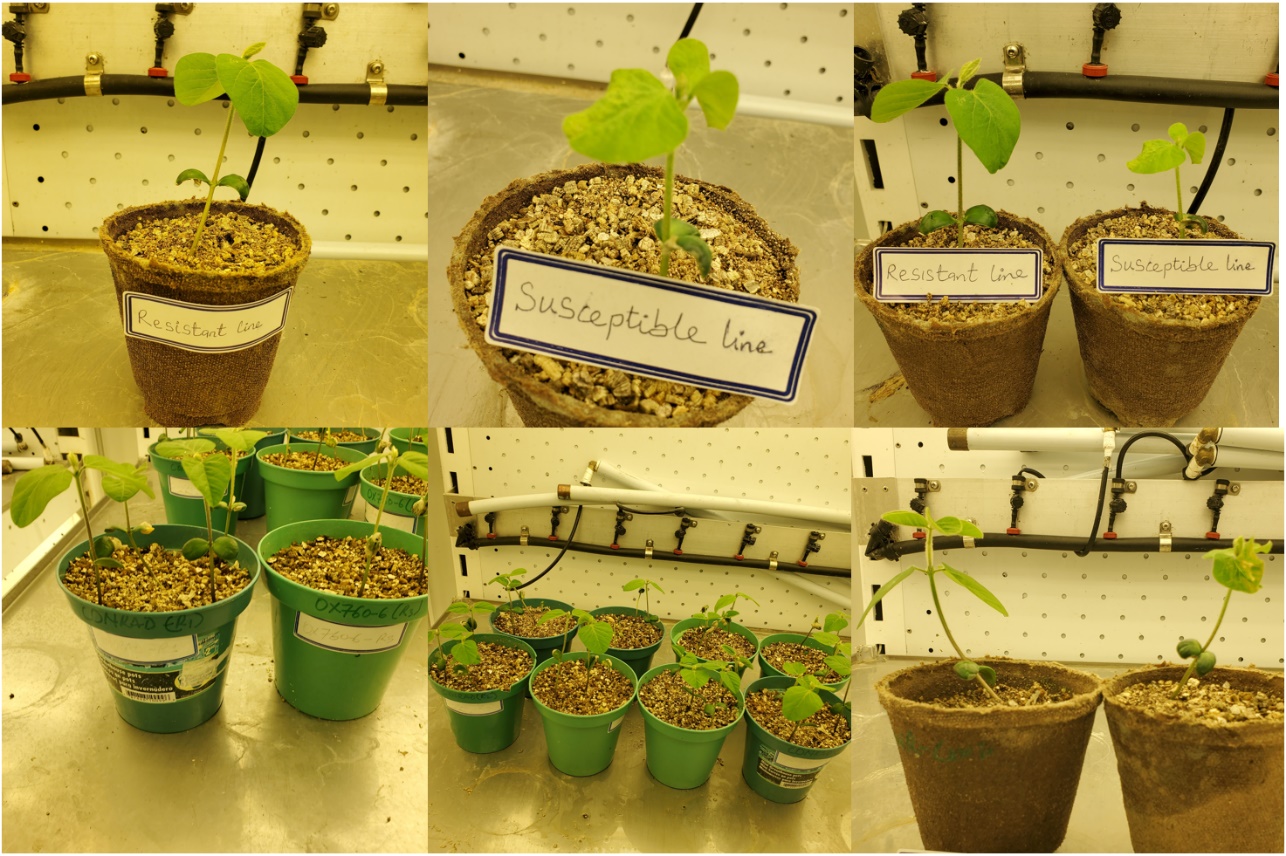


**Figure S1.** Soybean seedlings maintained under controlled conditions with 16 h of alternating light at 25^○^C and 8 h of dark at 20^○^C with relative humidity of 60% inside a growth chamber. Sterilized dH_2_O was applied every day to maintain the vermiculite water content from moist to slightly dry to provide optimum nutrients and moisture to seedlings.


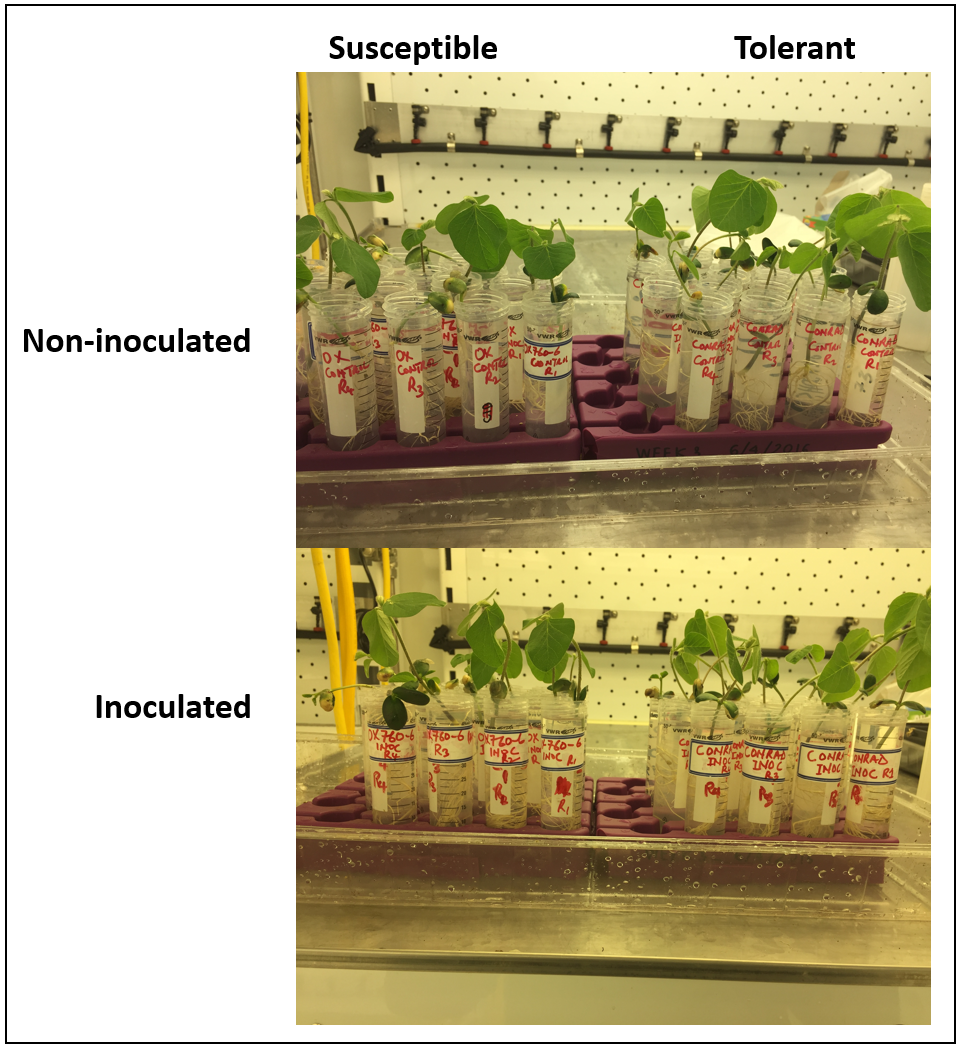


**Figure S2.** Soybean seedlings maintained under controlled conditions with 16 h of alternating light at 25^○^C and 8 h of dark at 20^○^C with relative humidity of 60% inside a growth chamber. Sterilized dH_2_O was applied every day to maintain the vermiculite water content from moist to slightly dry to provide optimum nutrients and moisture to seedlings. Seedlings of susceptible (OX760-6) cultivar and tolerant (CONRAD) cultivar were carefully removed from the pots and inoculated using zoospore method of inoculation.

**
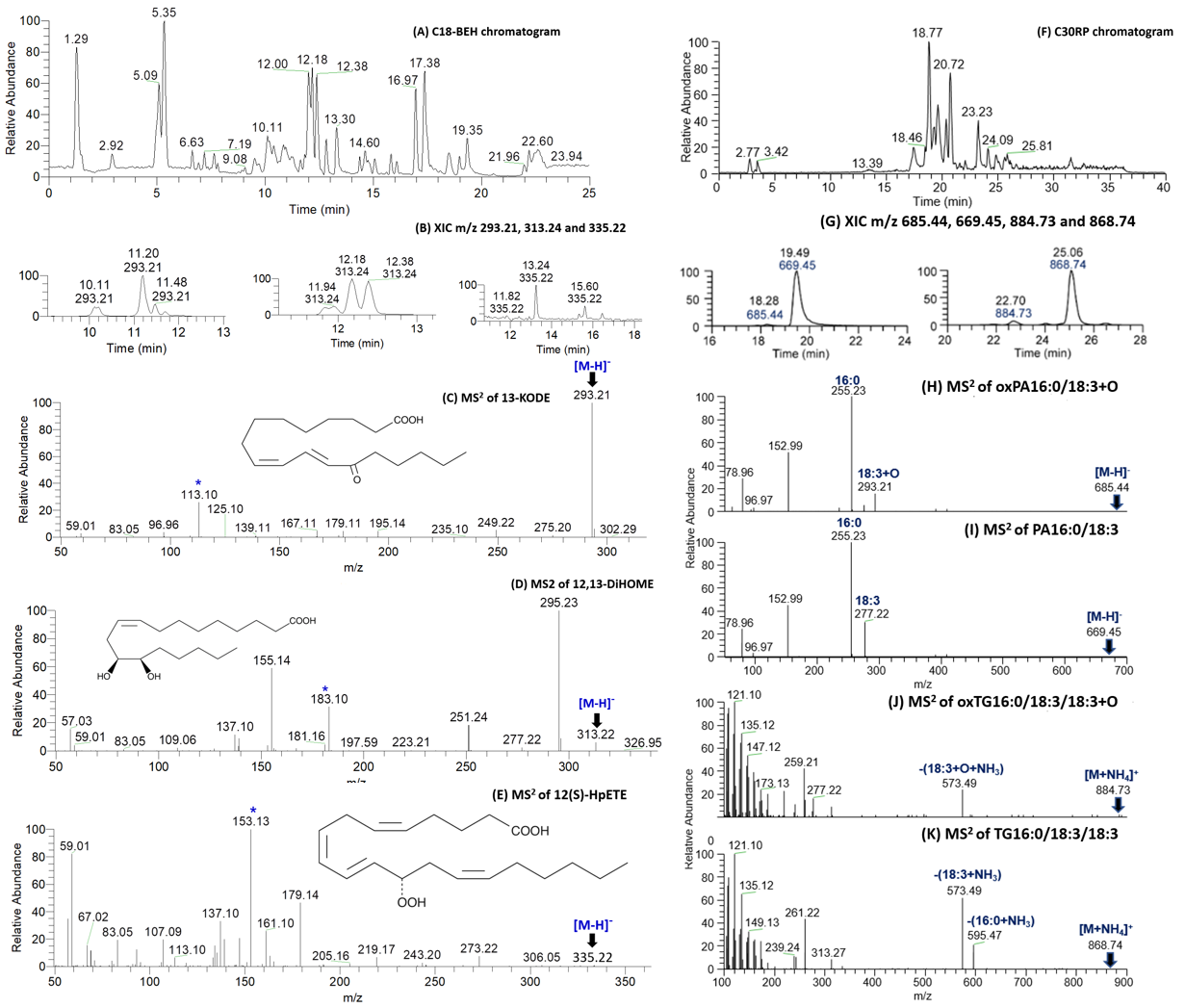
**

**Figure S3.** Chromatogram and mass spectrum of oxylipins observed in tolerant and susceptible soybean roots and stems in response to *P. sojae* infection. (A) Chromatogram demonstrating the C18-BEH-UHPLC-HRAM-MS/MS separation of primary oxylipins in the roots of both susceptible (OX760-6) and tolerant (Conrad) soybean cultivars infected with *P. sojae*; (B) Extracted ion chromatogram (XIC) of *m/z* 293.21, 313.24 and 335.22 precursor ions of the selected oxylipin compounds present in the inoculated root of both soybean cultivars in response to infection with *P. sojae*; (C) MS^2^ spectrum of *m/z* 293.21 identified as 13-KODE; (D) MS^2^ spectrum of *m/z* 313.24 identified as 12,13-DiHOME and (E) MS^2^ spectrum of *m/z* 335.22 identified as 12(S)-HpETE. The characteristic ions for identifying the different classes of oxylipins are marked with (*) and observed at *m/z* 113.10, 183.10 and 153.13 in (c-e), respectively; (F) UHPLC-C30RP-HESI-MS chromatogram showing oxidized intact glycerolipids in soybean roots following infection with *P. sojae*; (G) Extracted ion chromatogram (XIC) of precursor ions *m/z* 685.44, 669.45 in negative ion mode and *m/z* 884.73 and 868.74 in the positive ion mode of the selected oxidized /unoxidized) glycerolipids; (H, I) MS^2^ spectra of *m/z* 685.44 and 669.45 [M-H]^-^ precursor ions showing the presence of Ox-PA (PA16:0/18:3+O) in addition to the unoxidized PA 16:0/18:3 compound; and (J, K) MS^2^ spectra of *m/z* 884.73 and 868.74 [M+NH4]^+^ precursor ions showing the presence of Ox-TG (TG 16:0/18:3/18:3+O) in addition to the unoxidized version (TG 16:0/18:3/18:3). 13-KODE = (9Z,11E)-13-Oxo-9,11-octadecadienoic acid, 12,13-DiHOME = (*Z*)-12,13-dihydroxyoctadec-9-enoic acid, and 12(S)-HpETE = 12S-hydroperoxy-5(Z),8(Z),10(E),14(Z)-eicosatetraenoic acid, Ox-PA = oxidized phosphatidic acid, Ox-TG = oxidized triacylglycerol.

**
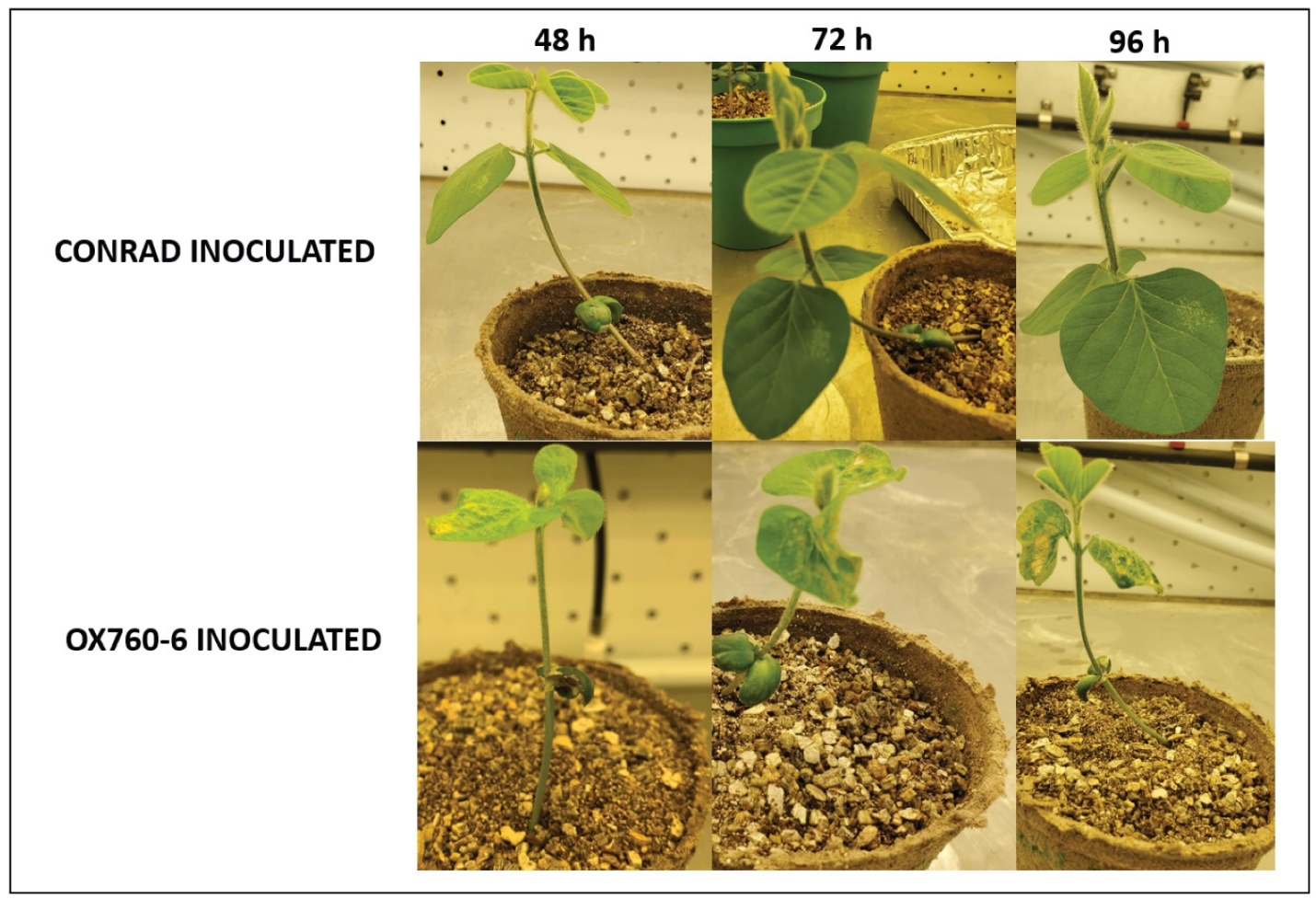
**

**Figure S4.** Soybean seedlings maintained under controlled conditions with 16 h of alternating light at 25^○^C and 8 h of dark at 20^○^C with relative humidity of 60% inside a growth chamber. Sterilized dH_2_O was applied every day to maintain the vermiculite water content from moist to slightly dry to provide optimum nutrients and moisture to seedlings. Seedlings of susceptible (OX760-6) cultivar and tolerant (CONRAD) cultivar were challenged with *P. sojae* infection for 48 h, 72 h and 96 h respectively.
